# Vitamin D alleviates indoxyl sulfate-induced inflammatory and cholesterol dysregulation in macrophages: Implications for vascular health in patients receiving hemodialysis

**DOI:** 10.1371/journal.pone.0357984

**Published:** 2026-09-11

**Authors:** Kyoung Hye Kong, Kyungwon Yang, Jung-Hwa Ryu

**Affiliations:** 1 Ewha Medical Research Center, Ewha Womans University College of Medicine, Seoul, Republic of Korea; 2 Inflammation-Cancer Microenvironment Research Center, Ewha Womans University College of Medicine, Seoul, Republic of Korea; 3 Department of Internal Medicine, Ewha Womans University College of Medicine, Seoul, Republic of Korea; Australian National University, AUSTRALIA

## Abstract

Indoxyl sulfate (IS) is a protein-bound uremic toxin that accumulates in patients with chronic kidney disease (CKD) and promotes oxidative stress, endothelial dysfunction, vascular smooth muscle cell proliferation, and fibrosis, thereby contributing to vascular stenosis in patients receiving hemodialysis. CKD is also commonly associated with vitamin D deficiency, which is linked to vascular calcification, immune dysregulation, and inflammation. This study aims to investigate the preventive effects of 1,25(OH)_2_D_3_ (active vitamin D) against IS-induced macrophage inflammatory activation and cholesterol dysregulation. Macrophages were pretreated with 30 nM 1,25(OH)_2_D_3_ for 12 and 24 h, followed by exposure to IS at concentrations of 125 and 250 µg/mL for 24 h. Morphological changes were observed under a microscope. To assess macrophage phenotype-associated changes, qPCR was performed to analyze the expression of M1-like/pro-inflammatory markers (TNF-α and IL-1β) and M2-like/anti-inflammatory phenotype-associated markers (CD163 TGF-β and IL-10). Cholesterol metabolism was assessed using a cholesterol efflux assay, qPCR analysis of ABCA1 and ABCG1 and Oil Red O staining for intracellular lipid accumulation. Protein expression of inflammatory mediators, TGF-β1, and cholesterol efflux transporters was further evaluated by western blotting. 1,25(OH)_2_D_3_ pretreatment modulated IS-associated inflammatory responses, as reflected by changes in selected M1-like/pro-inflammatory mediators, including iNOS, IL-6 and IL-1β. Conversely, 1,25(OH)_2_D_3_ increased selected M2-like/anti-inflammatory phenotype-associated markers, including CD163 and IL-10. Furthermore, 1,25(OH)_2_D_3_ pretreatment preserved cholesterol efflux capacity and modulated ABCA1 and ABCG1 expression in a time- and transporter-dependent manner, accompanied by reduced intracellular lipid accumulation as shown by Oil red O staining. These findings suggest that 1,25(OH)_2_D_3_ may protect macrophages against IS-induced inflammatory activation and cholesterol dysregulation, highlighting its potential as a preventive or modulatory approach for macrophage-mediated vascular dysfunction in CKD.

## Introduction

Chronic kidney disease (CKD) is a major public health concern owing to its high global prevalence and progressive nature. As renal function declines, the ability to eliminate metabolic waste products becomes impaired, leading to the systemic accumulation of uremic toxins [1,2]. Among these, protein-bound solutes such as indoxyl sulfate (IS) raise concern because they are poorly removed by conventional dialysis in advanced CKD and exhibit potent biological toxicity [3].

IS is a gut-derived metabolite of dietary tryptophan that is generated by the gut microbiota and subsequently processed through hepatic sulfation. IS has been implicated in the pathogenesis of cardiovascular complications in CKD and cardiovascular disease remains a leading cause of morbidity and mortality in this population [4,5]. Mechanistically, IS promotes reactive oxygen species generation, vascular smooth muscle cell proliferation, pro-inflammatory cytokine production, and endothelial dysfunction [6]. Collectively, these effects contribute to vascular remodeling and atherosclerosis, which are frequently observed in patients undergoing maintenance hemodialysis.

Vitamin D deficiency is a common feature of CKD, partly due to decreased renal 1α-hydroxylase activity, which impairs the conversion of 25-hydroxyvitamin D to its active form, 1,25-dihydroxyvitamin D3 [1,25(OH)_2_D_3_; active vitamin D] [7]. Beyond its classical role in calcium-phosphate metabolism and bone health, 1,25(OH)_2_D_3_ exerts pleiotropic effects on immune regulation and inflammation [8]. Vitamin D deficiency in CKD is associated with an increased cardiovascular risk, systemic inflammation, and immune dysregulation [9].

Macrophages play a central role in orchestrating vascular inflammation and remodeling. Depending on the local environmental signals, macrophages can be broadly polarized toward classically activated M1-like or alternatively activated M2-like phenotypes. M1-like macrophages produce pro-inflammatory mediators, such as tumor necrosis factor alpha (TNF-α) and interleukin-1β (IL-1β), thereby promoting tissue damage and atherogenesis. In contrast, M2-like macrophages express anti-inflammatory mediators, including IL-10 and CD163, which are associated with tissue repair and the resolution of inflammation [10].

IS promotes macrophage inflammatory activation and foam cell formation, thereby exacerbating lipid accumulation and vascular inflammation. These findings suggest a key role of macrophage dysfunction in IS-induced vascular pathology. Importantly, 1,25(OH)_2_D_3_ has been reported to modulate macrophage phenotype and cholesterol metabolism, including the upregulation of cholesterol efflux transporters such as ABCA1 and ABCG1. However, whether 1,25(OH)_2_D_3_ can prevent or attenuate IS-induced pro-inflammatory and proatherogenic macrophage dysfunction remains unclear [11,12].

This study aimed to elucidate whether pretreatment with 1,25(OH)_2_D_3_ protects macrophages against IS-induced inflammatory activation and cholesterol dysregulation. Specifically, we examined macrophage phenotype-associated markers, inflammatory gene expression, cholesterol efflux capacity, ABCA1/ABCG1 expression, and foam cell formation under IS-exposed conditions. These findings may provide mechanistic insight into the potential preventive or modulatory role of 1,25(OH)_2_D_3_ in macrophage-mediated vascular dysfunction in CKD.

## Materials and methods

### Isolation and culture of BMDMs

The animals were purchased from Orient BIO (Seongnam, Republic of Korea), and all animal procedures were approved by the Institutional Animal Care and Use Committee at the School of Medicine, Ewha Womans University (25−012). All methods were performed in accordance with relevant guidelines and regulations.

BMDMs were generated from bone marrow cells isolated from the femurs and tibias of male C57BL/6 mice (6–7 weeks old), as previously described with minor modifications. [13,14]. Mice were deeply anesthetized with isoflurane and euthanized by cervical dislocation. Bone marrow cells were flushed from the femurs and tibias using a 10-mL syringe fitted with a 25G needle containing Dulbecco’s modified eagle medium (DMEM) supplemented with 5% fetal bovine serum (FBS), 1% penicillin–streptomycin, and 1M 2-[4-(2-hydroxyethyl) piperazin-1-yl] ethanesulfonic acid (HEPES). The collected cell suspension was centrifuged at 1200 rpm for 3 min at room temperature (RT). The supernatant was discarded, and the pellet was resuspended in 1 mL of 10 mM ethylenediaminetetraacetic acid (EDTA) in phosphate-buffered saline (PBS). The volume was then adjusted to 25 mL with 10 mM EDTA/PBS solution and passed through a 100-µm cell strainer to remove debris. The filtrate was centrifuged again at 1200 rpm for 3 min at RT. After removing the supernatant, the pellet was resuspended in 5 mL of red blood cell lysis buffer and incubated at RT for 5 min to lyse the blood cells. Lysis was stopped by adding 35 mL of DMEM supplemented with 10% FBS-, followed by centrifugation at 1200 rpm for 3 min at RT. The supernatant was discarded, and the cell pellet was resuspended in 10 mL of DMEM supplemented with 10% FBS and transferred to a T75 flask. After incubation for 2–3 h to allow stromal cells to adhere, non-adherent cells were collected by gentle pipetting, centrifuged at 1200 rpm for 3 min at RT, counted, adjusted to a concentration of 1 × 10^4^ cells/mL, and cultured in complete medium (CM). CM consisted of DMEM containing 10% FBS, 1% penicillin–streptomycin, 1M HEPES, and 25 ng/mL granulocyte-macrophage colony-stimulating factor (GM-CSF; R&D Systems, Minneapolis, MN, USA). Cells were cultured at 37 °C in a humidified incubator with 5% CO_2_ throughout the differentiation process. On day 4, fresh CM was added. On day 7, adherent differentiated BMDMs were harvested and used for subsequent experiments.

### Experimental design and treatment groups

To evaluate the protective effects of 1,25(OH)_2_D_3_ (active vitamin D; Enzo Life Sciences, Farmingdale, NY, USA) against IS-induced macrophage dysfunction, differentiated BMDMs were divided into the following experimental groups: i) untreated BMDMs as the control group (M0), ii) BMDMs stimulated with LPS (100 ng/mL) and IFN-γ (20 ng/mL) (Sino Biological, Inc. Beijing, China) to induce M1 polarization (M1); iii) M1-polarized BMDMs pretreated with 30 nM 1,25(OH)_2_D_3_ for either 12 or 24 h; iv) M1-polarized BMDMs treated with IS (Sigma Chemical Co, St. Louis, MO) at 125 or 250 µg/mL for 12 or 24 h; and v) M1-polarized BMDMs pretreated with 30 nM 1,25(OH)_2_D_3_ for 12 or 24 h, followed by co-treatment with IS (125 or 250 µg/mL) for an additional 24 h. The cells were harvested at 36 or 48 h post-treatment, depending on the experimental endpoint. The IS concentrations of 125 and 250 μg/mL were selected as in vitro challenge concentrations based on previous studies demonstrating IS bioactivity in macrophages at comparable experimental ranges [15,16]. These concentrations were used to induce measurable and reproducible macrophage responses under controlled in vitro conditions, rather than to directly reproduce circulating free IS levels in patients receiving hemodialysis.

### Cell proliferation and cytotoxicity assay

To determine the effects of IS and 1,25(OH)_2_D_3_ on cell proliferation, viability, and cytotoxicity, BMDMs were seeded into 96-well plates at a density of 1 × 10^4^ cells/well. After treatment under each experimental condition, the following assays were performed. Cell proliferation was assessed using the CellTiter 96® Aqueous One Solution Cell Proliferation Assay (Promega, Madison, WI, USA) according to the manufacturer’s instructions. Following treatment, 20 µL of the MTS reagent was added directly to each well containing 100 µL of culture medium. Plates were incubated for 2 h at 37 °C in a humidified 5% CO_2_ incubator, and absorbance was measured at 490 nm using a microplate reader (Molecular Devices, San Jose, CA, USA). Cell viability and membrane integrity were assessed by measuring LDH release into the culture supernatant using a CytoTox96 Non-Radioactive Cytotoxicity Assay (Promega, Madison, WI, USA). After incubation with the appropriate substrates for 30 min at RT, the reaction was stopped according to the manufacturer’s instructions, and absorbance was measured at 490 nm. All experiments were conducted in triplicate, and the data were normalized to the untreated control (M0) group.

### Quantitative reverse transcriptase polymerase chain reaction (RT-qPCR) analysis

Total RNA was isolated from BMDMs using the AccuPrep® Universal RNA Extraction Kit (Bioneer, Daejeon, Republic of Korea), and cDNA was synthesized using CycleScript™ RT PreMix (dN6) (Bioneer, Daejeon, Republic of Korea). RT-qPCR was performed using amfiSure qGreen Q-PCR Master Mix (2×) and High ROX (GenDEPOT, Katy, TX, USA). Primer sequences were selected based on previously published sequences and checked for target specificity. The experiments were repeated three times. The RT-qPCR primers sequences were as follows: TNF-α, forward, 5’-CCT CTT CTC ATT CCT GCT TGT GG-3’ and reverse, 5’-GGC CAT TTG GGA ACT TCT CAT C-3’; IL-1β, forward, 5’-TCG AGG CCT AAT AGG CTC ATC T-3’ and reverse, 5’-GCT GCT TCA GAC ACT TGC ACA A-3’; IL-10, forward, 5’-ACC TGC TCC ACT GCC TTG CT-3’, and reverse, 5’-GGT TGC CAA GCC TTA TCG GA-3’ [17]; ABCA1, forward, 5’-ATA GCA GGC TCC AAC CCT GAC-3’ and reverse,5’-GGT ACT GAA GCA TGT TTC GAT GTT-3’ [18]; ABCG1, forward, 5’-CTA CGG CTT GGA CCG AGA AG-3’ and reverse, 5’-ACC TCT CAG CCC GGA TTT TG-3’ [19]; TGF-β1, forward, 5’-GAA AGC CCT GTA TTC CGT CTC CTT-3’, and reverse, 5’-CAA CAA TTC CTG GCG TTA CCT-3’ [20]; CD163, forward, 5’-GTC CTC CTC ATT GTC TTC CTC-3’ and reverse, 5’-ATC CGC CTT TGA ATC CAT CTC-3’ [21].

The expression levels of CD163, TNF-α, IL-1β, IL-10, TGF-β1, ABCA1, and ABCG1 were evaluated using a QuantStudio 3 Real-time PCR System (Applied Biosystems, Foster City, CA, USA). After normalization to hypoxanthine-guanine phosphoribosyltransferase (HPRT), a housekeeping gene, relative gene expression was calculated using the comparative Ct method (2^−ΔΔCT^).

### Cholesterol efflux assay

The cholesterol efflux capacity was measured using the Abcam Cholesterol Efflux Assay Kit (Cell-based; ab196985) according to the manufacturer’s protocol. Briefly, cells were seeded at a density of 1×10^5^ cells/well in a 96-well plate and incubated overnight at 37 °C in a humidified incubator with 5% CO_2_ to allow adherence.

The cells were then labeled with 100 µL of labeling medium, consisting of a 1:1 mixture of labeling reagent and phenol red-free, serum-free RPMI 1640 medium. After 1-hour incubation at 37 °C protected from light, the cells were washed and incubated overnight (12–16 h) with 100 µL/well of equilibration medium, prepared by mixing equilibration buffer (supplemented with reagent A [2 µL/mL] and reagent B [10 µL/mL]) and RPMI 1640 in a 1:1 ratio.

After equilibration, the cells were washed with RPMI 1640 medium and treated with IS and 1,25(OH)_2_D_3_ as described above in cholesterol acceptor-containing RPMI 1640 medium for 4 h at 37 °C. Human plasma-derived native HDL (Merck Millipore/Calbiochem; Cat. No. 437641, Darmstadt, Germany) was used as an exogenous cholesterol acceptor at a final concentration of 20 µg/mL. According to the manufacturer’s information, this HDL preparation was isolated from human plasma by sequential flotation ultracentrifugation and consisted of approximately 45–55% protein and 45–55% lipid. The source plasma was tested negative for HBsAg, HIV-1, HIV-2, HBc, and hepatitis C antibodies. For the positive control, 20 µL of the kit-supplied positive control reagent was added. Negative control wells were treated with serum-free RPMI 1640 medium alone.

At the end of the incubation, 100 µL of supernatant was collected and transferred to a white opaque 96-well plate, and fluorescence was measured using BioTek Synergy H1 microplate reader (Santa Clara, CA, USA) at Ex/Em = 485/523 nm. Cells were lysed in 100 µL of cell lysis buffer for 30 min at RT with orbital shaking, and the lysates were transferred to a white opaque 96-well plate for fluorescence detection. Background fluorescence was corrected using cell-free control wells according to the manufacturer’s protocol, and background values were subtracted from the experimental readings before cholesterol efflux calculation.

Cholesterol efflux was calculated as a percentage of total labeled cholesterol according to the following formula: Efflux (%) = [RFU of supernatant / (RFU of supernatant + RFU of cell lysate)] × 100. The final net cholesterol efflux was determined by subtracting the % cholesterol efflux value of the negative control from each treatment group, as recommended in the manufacturer’s protocol.

### Oil Red O staining

Lipid accumulation in BMDMs was evaluated using Oil Red O staining. Briefly, BMDMs were seeded into a 4-well cell culture slides (SPL, Gyeonggi-do, Republic of Korea) at 5 × 10^5^ cells/mL and treated under the indicated experimental conditions. After treatment, the cells were washed three times with 500 µL of PBS and then fixed with 500 µL of 4% formaldehyde for 30 min at RT. After removal of the fixation solution, the cells were washed twice with distilled water and rinsed with 60% (*v*/*v*) isopropanol for 5 min. The cells were then stained with 500 µL of Oil Red O working solution, prepared by mixing Oil Red O stock solution (Sigma Chemical Col, St. Louis, MO, USA) with distilled water at a ratio of 3:2, for 10 min at RT. After staining, the cells were rinsed three times with distilled water. Stained cells were covered with distilled water and examined under an inverted microscope (BX51; Olympus, Tokyo, Japan). Oil Red O staining was used to visualize intracellular lipid accumulation.

### Western blot analysis

Protein extraction and western blotting were performed as previously described [22,23]. Macrophages were washed with ice-cold PBS and lysed on ice using radioimmunoprecipitation assay lysis buffer (1×) supplemented with EDTA (GenDEPOT, Katy, TX, USA) and a complete protease inhibitor cocktail (GenDEPOT, Katy, TX, USA). Equal amounts of protein (50 µg) were separated by sodium dodecyl sulfate–polyacrylamide gel electrophoresis and transferred onto polyvinylidene fluoride membranes (Bio-Rad, Hercules, CA, USA). After transfer, the membranes were cut according to the expected molecular weight ranges of the target proteins before antibody incubation. The membranes were blocked with 5% nonfat dry milk and incubated with primary antibodies. The following primary antibodies were purchased from Abcam (Cambridge, United Kingdom): iNOS (ab3523), TGF-β1 (ab66043), IL-6 (ab290735), ABCG1 (ab52617), and ABCA1 (ab18180). The IL-1β antibody (12507) was purchased from Cell Signaling Technology (Danvers, MA, USA). β-actin was used as a loading control. The secondary antibodies used were horseradish peroxidase-conjugated goat anti-rabbit antibody (VectorLabs, USA) or goat anti-mouse antibody (GenDEPOT, Katy, TX, USA). Protein bands were detected using an enhanced chemiluminescence reagent (Thermo Scientific Pierce, Rockford, IL, USA) and luminescent Image Analyzer 3 (Bio-Rad, Hercules, CA, USA). Band densities were measured using ImageJ v.1.49 (NIH, Bethesda, MD, USA) and normalized to β-actin.

### Statistical analysis

Data are presented as the mean ± standard deviation or mean ± standard error of the mean, as indicated in each figure legend. Statistical analyses were performed using GraphPad Prism software version 8.0.1. Multiple-group comparisons were performed using one-way analysis of variance (ANOVA), followed by Tukey’s multiple comparisons test for comparisons among all groups or by Šídák’s multiple comparisons test for selected pairwise comparisons, as appropriate. Statistical significance was set at p < 0.05.

## Results

**Fig 1:**
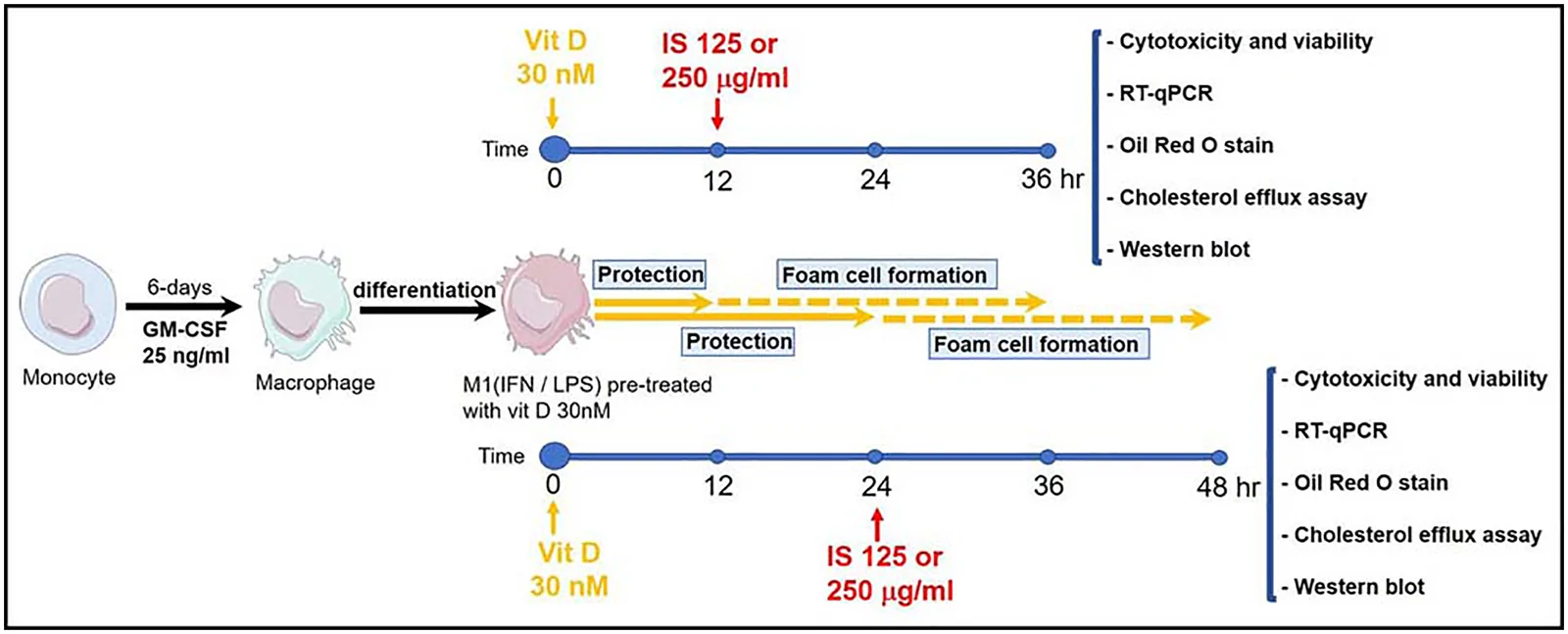
Experimental scheme for assessing the effects of 1,25(OH)_2_D_3_ on IS-associated macrophage dysfunction. Bone marrow-derived monocytes were differentiated into macrophages for 6 days in the presence of GM-CSF (25 ng/mL), followed by M1 polarization with LPS/IFN-γ. Cells were pretreated with 1,25(OH)_2_D_3_ (30 nM) before exposed to IS (125 or 250 µg/mL). Two experimental timelines were used: at 12 h 1,25(OH)_2_D_3_ pretreatment followed by IS exposure and endpoint analysis at 36 h (upper), and a 24 h 1,25(OH)_2_D_3_ pretreatment followed by IS exposure and endpoint analysis at 48 h (lower). The assessed endpoints included cytotoxicity and viability assays, RT-qPCR analysis, Oil Red O staining, cholesterol efflux assays, and western blotting. The schematic illustrates the experimental design used to evaluate the potential protective effects of 1,25(OH)_2_D_3_ against IS-associated inflammatory activation, lipid accumulation, and cholesterol efflux dysregulation in macrophage. IS, indoxyl sulfate; GM-CSF, granulocyte-macrophage colony-stimulating factor; LPS, lipopolysaccharide; IFN-γ, interferon gamma; RT-qPCR, quantitative reverse transcriptase polymerase chain reaction.

To determine whether 1,25(OH)_2_D_3_ protects macrophages against IS-associated inflammatory and cholesterol-related dysfunction, monocytes were differentiated into macrophages over 6 days using granulocyte-macrophage colony-stimulating factor (GM-CSF), followed by polarization toward the M1 phenotype with lipopolysaccharide (LPS)/interferon gamma (IFN-γ). 1,25(OH)_2_D_3_ (30 nM) was administered as a pretreatment before IS exposure at 125 or 250 µg/mL over two experimental timelines (12 or 24 h), with endpoints at 36 and 48 h, respectively (Fig 1).

### Effects of 1,25(OH)_2_D_3_ on cell viability and proliferation in IS-exposed M1-polarized macrophages

**Fig 2:**
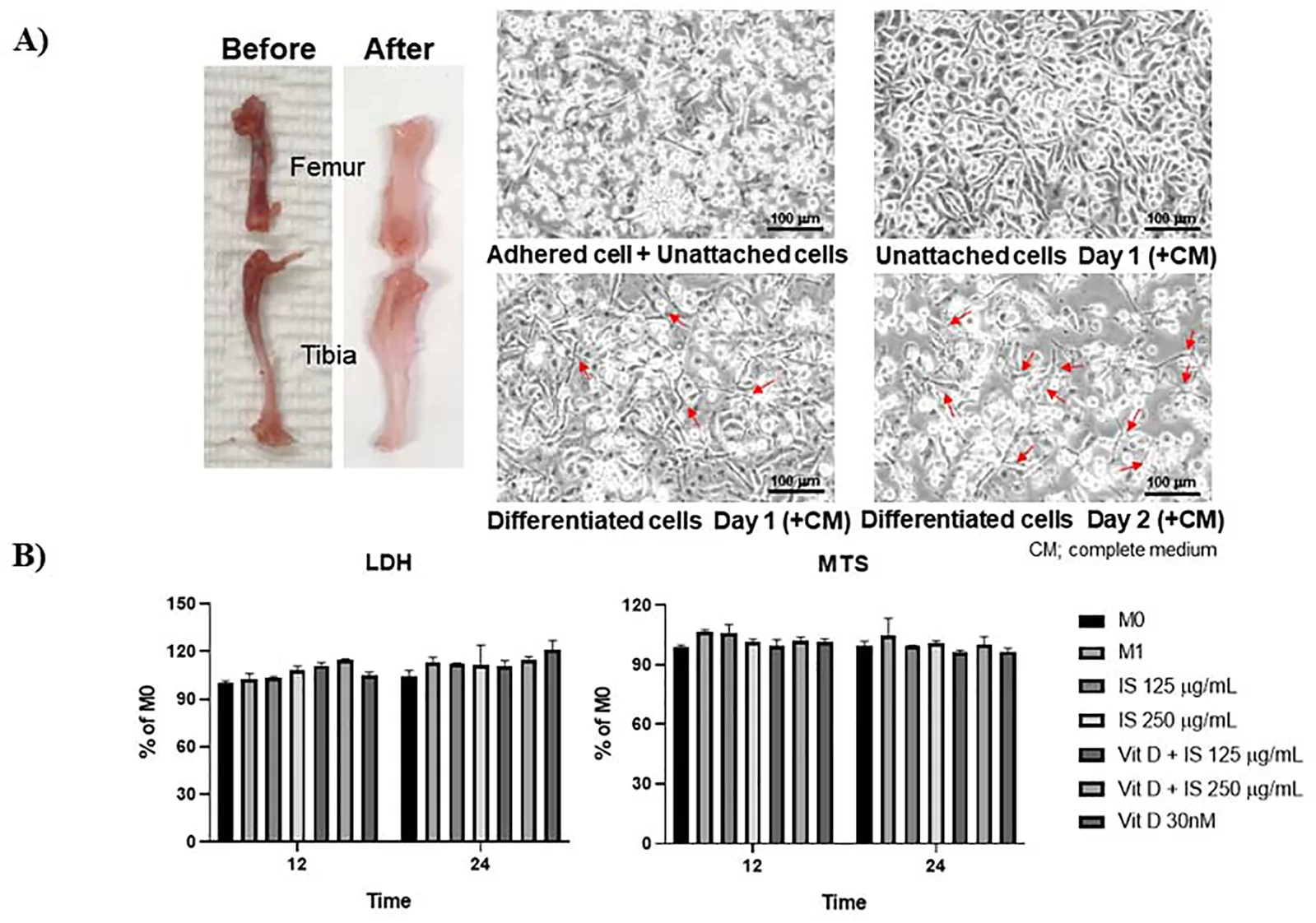
Isolation, differentiation, and viability assessment of bone marrow-derived macrophages (BMDMs). (A) Representative images showing murine femur and tibia before and after bone marrow flushing. Microscopic images show the early stages of BMDM culture and differentiation in complete medium (CM), including adhered and unattached cells, unattached cells on day 1, and differentiated macrophage-like cells on days 1 and 2. Red arrowheads indicate representative differentiated macrophage-like cells. Scale bars = 100 µm. (B) Cytotoxicity and cell viability were assessed by lactate dehydrogenase (LDH) release and MTS assays, respectively, after 12 and 24 h of treatment. The experimental groups included untreated macrophages (M0), LPS/IFN-γ-stimulated M1 macrophages (M1), IS-treated macrophages (125 or 250 µg/mL), 1,25(OH)_2_D_3_ -treated macrophages (30 nM), and macrophages pretreated with 1,25(OH)_2_D_3_ followed by IS exposure. The results are expressed as a percentage of the M0 control. Data are presented as means ± standard deviations. BMDMs, bone marrow-derived macrophages; CM, complete medium; IS, indoxyl sulfate; LPS, lipopolysaccharide; IFN-γ, interferon gamma; LDH, lactate dehydrogenase.

To establish bone marrow-derived macrophages (BMDMs), bone marrow cells were isolated from murine femurs and tibias and cultured in complete medium (CM). During the early differentiation period, the cells progressively exhibited macrophage-like morphological changes in CM (Fig 2A).

To determine whether IS or 1,25(OH)_2_D_3_ affected cell viability under the experimental conditions, lactate dehydrogenase (LDH) release and 3-(4,5-dimethylthiazol-2-yl)-2,5-diphenyltetrazolium bromide assay (MTS) assays were performed. Compared with the untreated macrophage control (M0), IS treatment at 125 or 250 µg/mL and 1,25(OH)_2_D_3_ treatment at 30 nM, either alone or in combination, did not markedly increase LDH release or reduce MTS activity at 12 and 24 h. Similarly, LPS/IFN-γ-stimulated M1 macrophages showed comparable LDH release and MTS activity. These findings indicate that neither IS nor 1,25(OH)_2_D_3_ compromised cell viability under the experimental conditions used (Fig 2B).

### 1,25(OH)_2_D_3_ modulated inflammatory phenotype-associated gene expression and preserved cholesterol-related gene expression in IS-exposed M1-polarized macrophages

**Fig 3:**
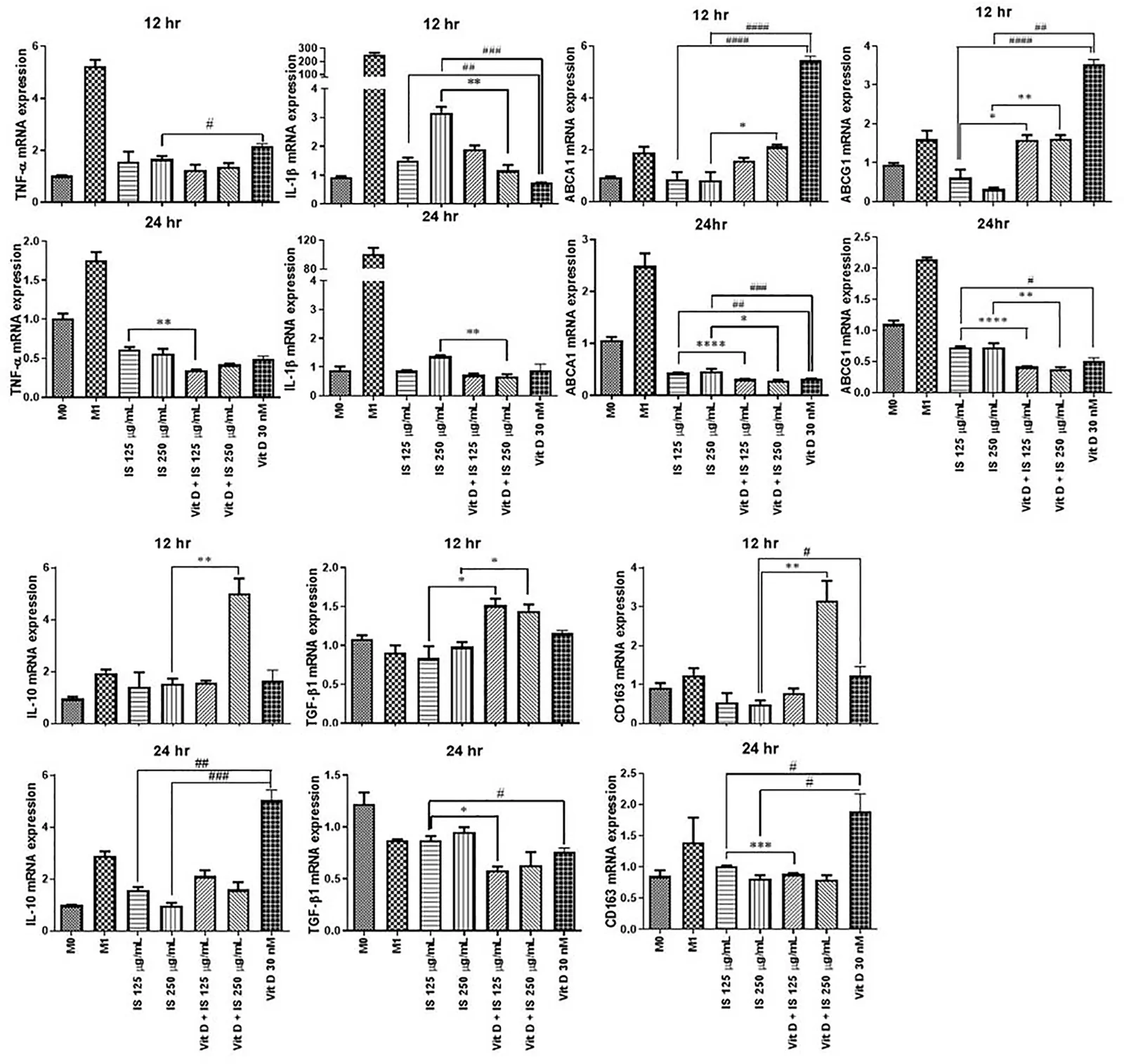
Effects of IS and 1,25(OH)_2_D_3_ on M1-like/pro-inflammatory, cholesterol efflux-related, and M2-like/anti-inflammatory phenotype-associated gene expression in BMDMs. The mRNA expression levels of M1-like/pro-inflammatory mediators (TNF-α and IL-1β), cholesterol efflux-related transporters (ABCA1 and ABCG1), and M2-like/anti-inflammatory phenotype-associated markers (IL-10, TGF-β1, and CD163) were assessed by RT-qPCR at 12 and 24 h. LPS/IFN-γ-stimulated M1 macrophages showed increased TNF-α and IL-1β expression compared with untreated M0. Under IS-exposed conditions, 1,25(OH)_2_D_3_ pretreatment modulated IL-1β mRNA expression, whereas its effect on TNF-α mRNA expression was relatively modest. ABCA1 and ABCG1 mRNA expression decreased in M1-polarized macrophages, whereas 1,25(OH)_2_D_3_ pretreatment preserved their expression under selected IS-exposed conditions, with a more apparent effect at 12 h. IL-10 and CD163 expression were increased by 1,25(OH)_2_D_3_ treatment at selected time points, whereas TGF-β1 showed no consistent increase. Data are expressed as means ± standard deviations. Statistical significance was assessed using one-way ANOVA followed by Tukey’s or Šídák’s multiple comparisons test, as appropriate. *p < 0.05, **p < 0.01, ***p < 0.001, ****p < 0.0001 versus the indicated groups; ^#^p < 0.05, ^##^p < 0.01, ^###^p < 0.001, ^####^p < 0.0001 between specific comparisons. IS, indoxyl sulfate; BMDMs, bone marrow-derived macrophages; LPS, lipopolysaccharide; IFN-γ, interferon gamma; RT-qPCR, quantitative reverse transcriptase polymerase chain reaction.

The mRNA expression levels of M1-like/pro-inflammatory markers, cholesterol efflux-related transporters, and M2-like/anti-inflammatory phenotype-associated markers were assessed in BMDMs at 12 and 24 h (Fig 3).

LPS + IFN-γ-stimulated M1 macrophages exhibited increased TNF-α and IL-1β mRNA expression compared with M0 controls at both time points. However, IS treatment in M1-polarized macrophages did not further increase TNF-α and IL-1β mRNA expression; rather, these cytokine transcripts were lower than those in M1-polarized macrophages under some conditions. Under selected IS-exposed conditions, 1,25(OH)_2_D_3_ pretreatment modulated IL-1β mRNA expression, whereas its effect on TNF-α mRNA expression was relatively modest.

The expression of the cholesterol efflux transporters ABCA1 and ABCG1 was reduced in M1-polarized macrophages, whereas 1,25(OH)_2_D_3_ pretreatment preserved ABCA1 and ABCG1 expression under selected IS-exposed conditions. This effect was more apparent at 12 h than at 24 h.

Among M2-like/anti-inflammatory phenotype-associated markers, IL-10 expression was increased by 1,25(OH)_2_D_3_ treatment, and CD163 was also upregulated by 1,25(OH)_2_D_3_ at selected time points. In contrast, TGF-β1 showed no significant change.

Together, these findings suggest that1,25(OH)_2_D_3_ pretreatment modulates inflammatory gene expression and preserves cholesterol efflux-related gene expression in IS-exposed macrophages in a marker- and time-dependent manner.

### 1,25(OH)_2_D_3_ reduced lipid accumulation in IS-exposed M1-polarized macrophages

**Fig 4:**
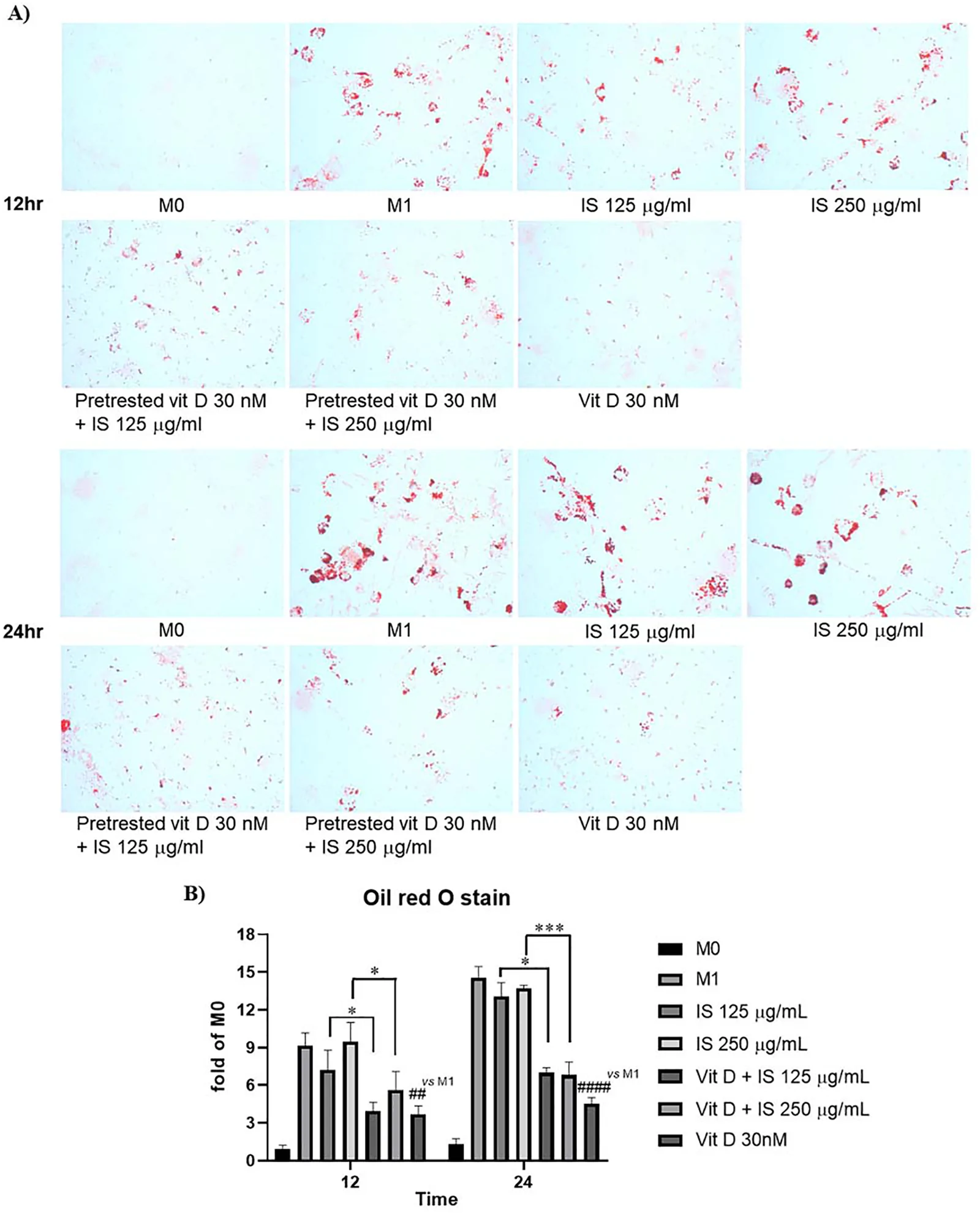
Effects of 1,25(OH)_2_D_3_ on lipid accumulation in IS-exposed BMDMs. (A) Representative full-color Oil Red O staining images of BMDMs under the indicated treatment conditions at 12 and 24 h. The images show intracellular lipid accumulation across experimental groups. (B) Quantitative analysis of Oil Red O staining intensity, expressed relative to the untreated macrophages control (M0), was performed to assess lipid accumulation. Data are presented as means ± standard deviations from at least three independent experiments. Statistical significance was assessed using one-way ANOVA followed by Tukey’s or Šídák’s multiple comparisons test, as appropriate. *p < 0.05, ***p < 0.001 versus indicated groups; ^##^p < 0.01, ^###^p < 0.001 versus M1. IS, indoxyl sulfate; BMDMs, bone marrow-derived macrophages.

Oil Red O staining was performed to assess intracellular lipid accumulation in BMDMs under the indicated treatment conditions (Fig 4A). M1-polarized macrophages exhibited increased lipid droplet formation at 12 and 24 h compared with the M0 controls. Pretreatment with 30 nM 1,25(OH)_2_D_3_ attenuated lipid accumulation in IS-exposed cells. Quantitative analysis confirmed these findings, showing increased Oil Red O staining intensity in M1-polarized macrophages, which was partially reduced by 1,25(OH)_2_D_3_ pretreatment under IS-exposed conditions (Fig 4B).

These results suggest that 1,25(OH)_2_D_3_ pretreatment attenuates lipid accumulation in IS-exposed macrophages under pro-inflammatory conditions.

### 1,25(OH)_2_D_3_ preserved cholesterol efflux capacity in IS-exposed M1-polarized macrophages

**Fig 5:**
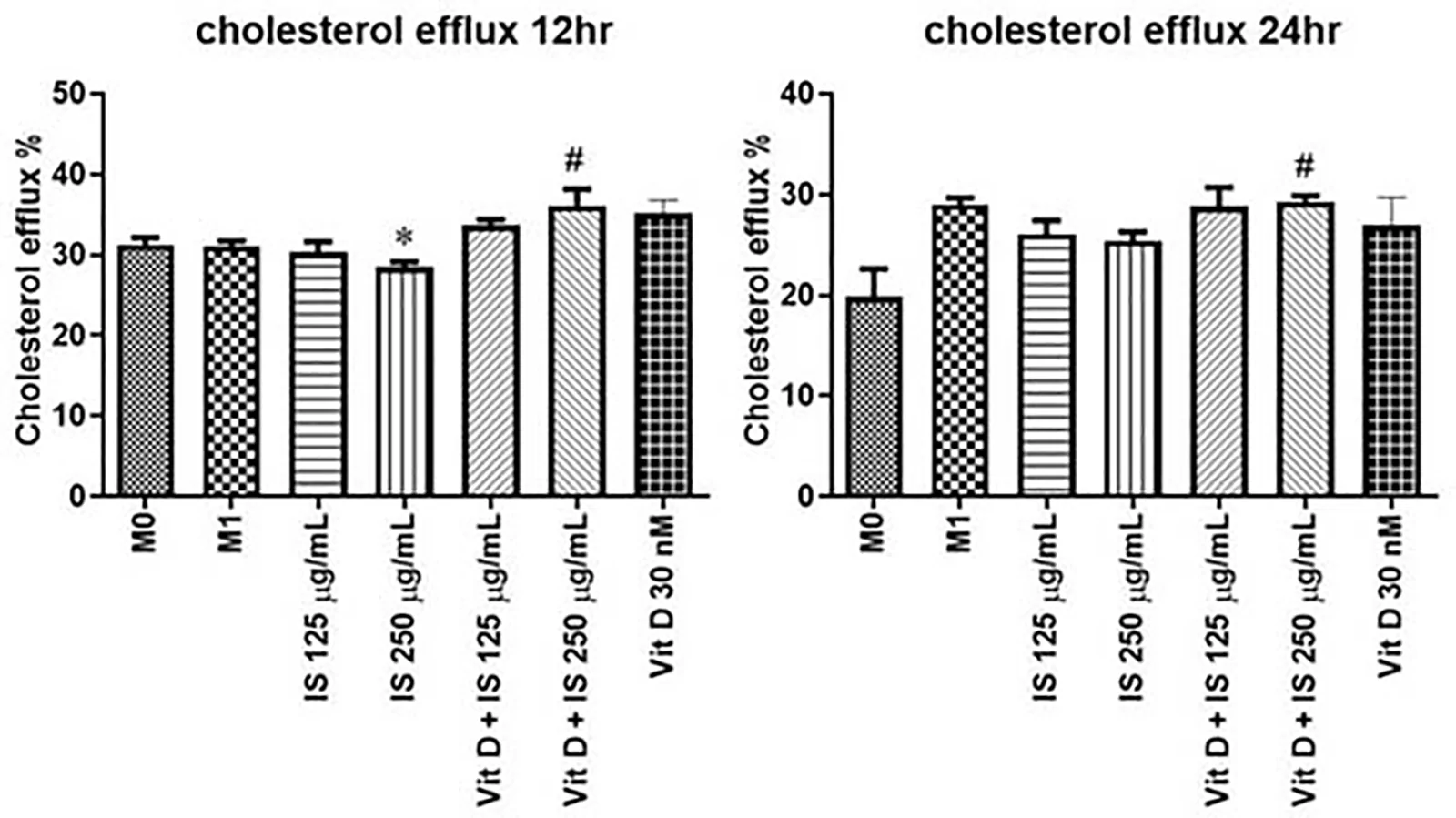
Effects of 1,25(OH)_2_D_3_ on cholesterol efflux capacity in IS exposed BMDMs. Cholesterol efflux was measured in BMDMs under the indicated treatment conditions at 12 (left) and 24 h (right). At 12 h, IS exposure at 250 µg/mL reduced cholesterol efflux capacity under selected IS-exposed conditions. At 24 h, IS exposure showed a modest effect on cholesterol efflux, and 1,25(OH)_2_D_3_ pretreatment increased or preserved cholesterol efflux capacity in IS-exposed macrophages. Data are presented as means ± standard deviations from at least three independent experiments. Statistical significance was assessed using one-way ANOVA followed by Tukey’s or Šídák’s multiple comparisons test, as appropriate. *p < 0.05 versus M1; ^#^p < 0.05 versus the corresponding IS alone group. IS, indoxyl sulfate; BMDMs, bone marrow-derived macrophages.

Cholesterol efflux capacity was evaluated in BMDMs under the indicated treatment conditions at 12 and 24 h (Fig 5). At 12 h, IS exposure at 250 µg/mL reduced cholesterol efflux compared with M1 controls, whereas 125 µg/mL showed no substantial effect. Pretreatment with 1,25(OH)_2_D_3_ preserved cholesterol efflux capacity in IS-exposed macrophages, with the 1,25(OH)_2_D_3_ + IS (125 µg/mL) group showing higher efflux than the corresponding IS alone group.

A similar pattern was observed at 24 h. IS exposure slightly decreased cholesterol efflux compared with M1 macrophages, although this difference was not statistically significant. 1,25(OH)_2_D_3_ pretreatment preserved or increased cholesterol efflux capacity in IS-exposed macrophages compared with IS-treated cells without 1,25(OH)_2_D_3_. In addition, 1,25(OH)_2_D_3_ alone maintained cholesterol efflux at levels comparable to those of M1 controls at both time points.

Together, these findings suggest that 1,25(OH)_2_D_3_ pretreatment helps preserve macrophage cholesterol efflux capacity under IS-exposed conditions.

### 1,25(OH)_2_D_3_ differentially modulated inflammatory mediator and cholesterol efflux transporter protein expression in IS-exposed macrophages

**Fig 6:**
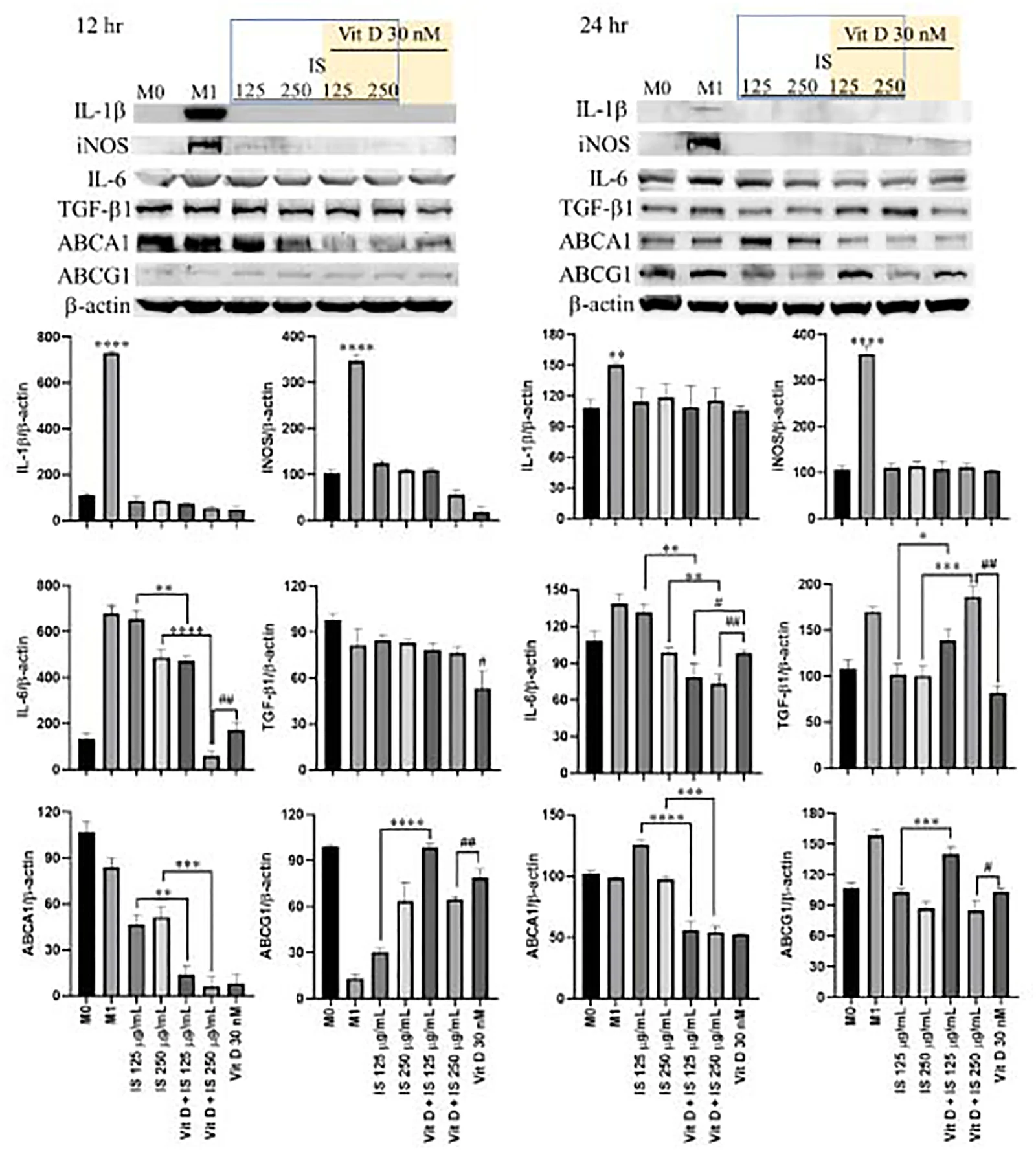
Effects of 1,25(OH)_2_D_3_ on M1-like/pro-inflammatory mediators and cholesterol efflux transporter protein expression in IS-exposed macrophages. Representative immunoblots and quantitative analyses of M1-like/pro-inflammatory mediators (IL-1β, iNOS, and IL-6), TGF-β1, and cholesterol efflux transporters (ABCA1 and ABCG1) in M0 and M1 macrophages treated with IS (125 and 250 µg/mL) in the absence or presence of 1,25(OH)_2_D_3_ (30 nM) for 12 (left) and 24 h (right). β-Actin was used as a loading control. 1,25(OH)_2_D_3_ modulated selected M1-like/pro-inflammatory mediators in a marker- and time-dependent manner. ABCA1 and ABCG1 showed transporter-specific responses, with ABCG1 being better maintained under selected 1,25(OH)_2_D_3_-treated conditions, whereas ABCA1 was not uniformly preserved. Data are presented as means ± standard errors of the mean (n = 3–4). Statistical significance was determined using a one-way ANOVA followed by Tukey’s or Šídák’s multiple comparisons test, as appropriate. *p < 0.05, **p < 0.01, ***p < 0.001, ****p < 0.0001 versus IS alone; ^#^p < 0.05, ^##^p < 0.01 versus 1,25(OH)_2_D_3_ 30 nM. IS, indoxyl sulfate; iNOS, inducible nitric oxide synthase; BMDMs, bone marrow-derived macrophages.

To further evaluate the effects of 1,25(OH)_2_D_3_ on IS-associated inflammatory and cholesterol-related alterations at the protein level, M1-like/pro-inflammatory mediators, TGF-β, and cholesterol efflux transporters were examined by western blotting. The analyzed M1-like/pro-inflammatory mediators included IL-1β, iNOS, and IL-6 and the cholesterol efflux transporters included ABCA1 and ABCG1 (Fig 6).

At 12 h, M1 macrophages showed increased IL-1β, iNOS, and IL-6 protein expression compared with M0 cells. Under IS-exposed conditions, 1,25(OH)_2_D_3_ pretreatment differentially modulated selected M1-like/inflammatory mediators. IL-6 showed treatment-dependent changes, whereas IL-1β and iNOS exhibited distinct treatment- and time-dependent expression patterns. TGF-β1 expression also varied according to treatment condition. In parallel, IS exposure altered ABCA1 and ABCG1 protein expression. However, the effects of 1,25(OH)_2_D_3_ on these cholesterol efflux transporters were transporter-dependent; ABCA1 was not consistently preserved, whereas ABCG1 was maintained or increased under selected 1,25(OH)_2_D_3_-treated conditions (Fig 6, left).

At 24 h, IL-1β and IL-6 protein levels were lower than those observed at 12 h but remained detectable in M1 and IS-treated macrophages. 1,25(OH)_2_D_3_ treatment continued to differentially modulate selected M1-like/pro-inflammatory mediators, although the direction and magnitude of these changes varied depending on the marker. TGF-β1 expression showed a treatment-dependent pattern, and 1,25(OH)_2_D_3_ also affected ABCA1 and ABCG1 protein expression under IS-exposed conditions (Fig 6, right). Notably, ABCG1 expression was better maintained under selected 1,25(OH)_2_D_3_-treated conditions, whereas ABCA1 showed a less consistent pattern across IS concentrations and time points.

Overall, these protein data indicate that 1,25(OH)_2_D_3_ does not uniformly suppress all inflammatory markers or uniformly preserve all cholesterol efflux transporters. Rather, 1,25(OH)_2_D_3_ modulates M1-like/pro-inflammatory mediator expression and cholesterol efflux transporter expression in a marker-, time- and transporter-dependent manner.

## Discussion

In this study, we demonstrated that pretreatment with 1,25(OH)_2_D_3_ modulated IS-associated inflammatory and lipid-related changes in macrophages. IS is a protein-bound uremic toxin that induces oxidative stress, vascular smooth muscle cell proliferation, and macrophage-mediated inflammation, thereby contributing to vascular stenosis and atherosclerosis in patients with CKD [3,6,12]. Our findings provide mechanistic insights into how 1,25(OH)_2_D_3_ modulates macrophage phenotype-associated markers, attenuates selected M1-like/pro-inflammatory mediators, and preserves cholesterol efflux capacity under IS-exposed conditions.

1,25(OH)_2_D_3_ pretreatment attenuated selected inflammatory responses in IS-exposed macrophages. IS exposure was associated with changes in selected M1-like/pro-inflammatory mediators, including IL-1β, iNOS, and IL-6, in a marker- and time-dependent manner, consistent with previous studies linking IS to macrophage-driven vascular inflammation [12,15]. Pretreatment with 1,25(OH)_2_D_3_ reduced selected M1-like/pro-inflammatory mediators while increasing M2-like/anti-inflammatory phenotype-associated markers, such as IL-10 and CD163. These findings suggest that 1,25(OH)_2_D_3_ modulates macrophage inflammatory responses and phenotype-associated marker expression under IS-exposed conditions, rather than uniformly suppressing all inflammatory markers. Moreover, 1,25(OH)_2_D_3_ exerted a protective effect on macrophage cholesterol homeostasis. IS exposure associated with reduced ABCA1/ABCG1 expression and cholesterol efflux capacity, accompanied by increased intracellular lipid accumulation. 1,25(OH)_2_D_3_ pretreatment preserved cholesterol efflux transporter expression and cholesterol efflux activity, accompanied by reduced intracellular lipid accumulation. These findings support the potential role of 1,25(OH)_2_D_3_ as a regulator of cholesterol efflux-related transporters involved in reverse cholesterol transport [24,25] Thus, the coordinated preservation of ABCA1 and ABCG1 expression by 1,25(OH)_2_D_3_ may represent an important mechanism by which it helps limit IS-associated lipid dysregulation and macrophage-derived vascular injury in CKD [3,4].

In addition, our study highlights the differential regulation of inflammatory pathways. Although 1,25(OH)_2_D_3_ reduced selected M1-like/pro-inflammatory mediators, including IL-1β and IL-6, its effect on TNF-α was relatively modest. This selective modulation suggests that 1,25(OH)_2_D_3_ may exert pathway-specific regulatory effects, possibly involving NF-κB- or MAPK-dependent mechanisms. However, these pathways were not directly examined in the present study and should therefore be validated in future mechanistic studies. The relationship between inflammatory cytokine mRNA and protein expression should also be interpreted carefully. In particular, IL-1β showed a non-parallel pattern between mRNA expression and intracellular protein levels in the present study. This may be explained by the complex regulation of IL-1β, which is controlled not only by transcriptional induction but also by post-transcriptional regulation, protein translation, proteolytic processing, secretion, and protein stability. Because pro-IL-1β requires proteolytic processing through inflammasome/caspase-1-related pathways to generate mature IL-1β, changes in IL-1β mRNA may not directly correspond to intracellular IL-1β protein levels at the same time point [26]. Previous studies have shown that vitamin D/VDR signaling can negatively regulate NLRP3 inflammasome activation and IL-1β secretion, suggesting that 1,25(OH)_2_D_3_ may modulate IL-1β regulation beyond transcriptional control [27]. Interestingly, we found that the effect of 1,25(OH)2D3 on cholesterol efflux transporter expression was time-dependent and transporter-specific. The preservation of ABCG1 expression was more evident at 24 h, whereas ABCA1 showed a less consistent pattern across IS concentrations and time points. The more pronounced preservation of ABCG1 at 24 h may reflect delayed or indirect regulation of cholesterol efflux transporter expression by 1,25(OH)_2_D_3_/VDR-related signaling, as previous studies have shown that 1,25(OH)_2_D_3_ can regulate LXR, ABCA1, and ABCG1 expression and promote cholesterol efflux in macrophage-derived foam cells [28]. In addition, transporter-specific post-translational mechanisms, including differences in protein stability and turnover, may also contribute to the non-parallel responses observed between ABCA1 and ABCG1. Future studies should investigate whether these pathway-specific effects are directly linked to inflammatory signaling pathways, ABCA1/ABCG1 transcriptional regulation, or indirect effects secondary to reduced oxidative stress.

These findings may have potential translational relevance. Clinical studies have shown that higher circulating IS levels are associated with adverse outcomes, including all-cause and cardiovascular mortality, in patients receiving hemodialysis [29,30]. In parallel, vitamin D deficiency is common in patients with CKD and those receiving hemodialysis and has been associated with increased cardiovascular morbidity and mortality in observational studies [31]. Together with these clinical observations, our in vitro findings suggest a possible mechanistic link by which 1,25(OH)_2_D_3_ may modulate macrophage inflammatory responses and cholesterol handling under IS-exposed conditions. However, further in vivo and clinical studies are required to determine whether these macrophage-level effects translate into improved vascular outcomes in patients with CKD or those receiving hemodialysis.

This study had several limitations. First, because the present study was performed using murine bone marrow-derived macrophages in vitro, the findings may not fully recapitulate the complex in vivo CKD environment or macrophage responses in patients with CKD or those receiving hemodialysis. Future studies using human macrophages, including monocyte-derived macrophages from patients receiving hemodialysis, would be valuable to validate whether the modulatory effects of 1,25(OH)_2_D_3_ observed in this study are reproduced in a clinically relevant cellular context. Second, the IS concentrations used in this study, 125 and 250 µg/mL, may exceed clinically observed IS exposure levels, particularly the circulating free IS fraction. Clinically observed serum total IS concentrations in patients receiving hemodialysis are commonly reported to be approximately 20–100 µg/mL, although higher levels have been reported in some patients [29,32]. For example, Yamamoto et al. reported a median total IS concentration of 31.6 µg/mL (IQR, 22.6–42.0 µg/mL), and Lin et al. reported an upper tertile range of 57.9–141.4 µg/mL in hemodialysis patients [32]. However, because IS is highly protein-bound, the circulating free IS fraction is substantially lower than the total IS concentration [3,33]. Therefore, the present findings should be interpreted as mechanistic in vitro evidence obtained under experimental challenge conditions, rather than as direct clinical dose-equivalent effects. However, the IS concentrations of 125 and 250 μg/mL were selected based on prior studies demonstrating IS bioactivity in macrophages at comparable experimental ranges. Adesso et al. showed that IS at 62.5–1000 μM enhanced LPS-induced inflammatory responses in J774A.1 macrophage, including nitric oxide release, iNOS and COX-2 expression, and TNF-α and IL-6 production [15]. Wakamatsu et al. further demonstrated that IS promoted IL-1β production in THP-1-derived macrophages through activation of AhR/NF-κB/MAPK signaling pathways [11]. In addition, Ribeiro et al. used IS at 60–200 µg/mL to simulate uremic conditions and reported macrophage dysfunction, ROS generation, and low-grade inflammatory responses [16]. Nakano et al. also demonstrated that IS promotes proinflammatory macrophage activation through OATP2B1 and Dll4-Notch signaling. Based on these prior studies, we selected 125 and 250 µg/mL as in vitro challenge concentrations to induce measurable and reproducible macrophage responses under controlled experimental conditions [12,15,16,32]. Future studies using clinically relevant total and free IS concentrations and albumin-containing culture systems are needed to validate the translational relevance of these findings. Third, the precise molecular mechanisms by which 1,25(OH)_2_D_3_ regulates ABCA1 and ABCG1 expression require further elucidation. Potential mechanisms may include vitamin D receptor-dependent transcriptional regulation, post-translational modulation, or indirect effects secondary to reduced oxidative stress [24,34]. In addition, we did not directly examine downstream signaling pathways, such as the aryl hydrocarbon receptor signaling, NF-κB/MAPK activation, or NLRP3 inflammasome activation, which have been implicated in IS-associated macrophage inflammatory responses [12,35]. Therefore, the complex interplay among IS, 1,25(OH)_2_D_3_, macrophages, and vascular tissues in vivo remains unclear. Further studies using in vivo CKD models, pathway-specific experiments, and multi-omics approaches are needed to validate these findings and establish causality.

In conclusion, 1,25(OH)_2_D_3_ pretreatment attenuated IS-associated macrophage inflammation and lipid dysregulation by preserving cholesterol efflux capacity, maintaining cholesterol efflux transporter expression, and increasing M2-like/anti-inflammatory phenotype-associated marker expression. These results suggest that 1,25(OH)_2_D_3_ may serve as a potential preventive or modulatory approach for macrophage-mediated vascular dysfunction in CKD. Further studies using clinically relevant IS concentrations, human macrophages, and in vivo CKD models are needed to determine the translational relevance of these findings.

## Supporting information

S1 File Original uncropped and unadjusted western blot images for Fig 6. This file contains the original uncropped and unadjusted western blot images corresponding to the western blot data presented in Fig 6.
(PDF)

S2 File Proposed schematic model of 1,25(OH)_2_D_3_ action against IS-induced macrophage dysfunction. IS exposure in CKD/hemodialysis is proposed to activate AhR-, ROS-, and NF-κB-related inflammatory signaling in macrophages, contributing to increased inflammatory mediators, reduced ABCA1/ABCG1 expression, impaired cholesterol efflux, and lipid accumulation. 1,25(OH)_2_D_3_ pretreatment activates VDR and may modulate IS-associated inflammatory signaling, preserve ABCA1/ABCG1 expression, maintain cholesterol efflux, and reduce lipid accumulation. Dashed arrows indicate proposed mechanisms or pathway interactions that were not directly tested in the present study. IS, indoxyl sulfate; VDR, vitamin D receptor; AhR, aryl hydrocarbon receptor; ROS, reactive oxygen species; NF-κB, nuclear factor-kappa B.
(JPG)
