# Supplementary material for: Vitamin D alleviates indoxyl sulfate-induced inflammatory and cholesterol dysregulation in macrophages: Implications for vascular health in patients receiving hemodialysis
Source: PLoS One. 2026 Sep 11;21(9):e0357984. doi: 10.1371/journal.pone.0357984 (PMC13567749; doi:10.1371/journal.pone.0357984)
Supplement: Supplementary file 1 [file pone.0357984.s001.pdf]

Fig 6_ raw western blot images

12hr

* Loading: ①M0 ②M1 ③ IS125 ④IS250 ⑤ IS125+vitD 30nM ⑥ IS250+vitD 30nM ⑦vitD 30nM

IL-1β

Technical replicate #1 (used in Figure 6)

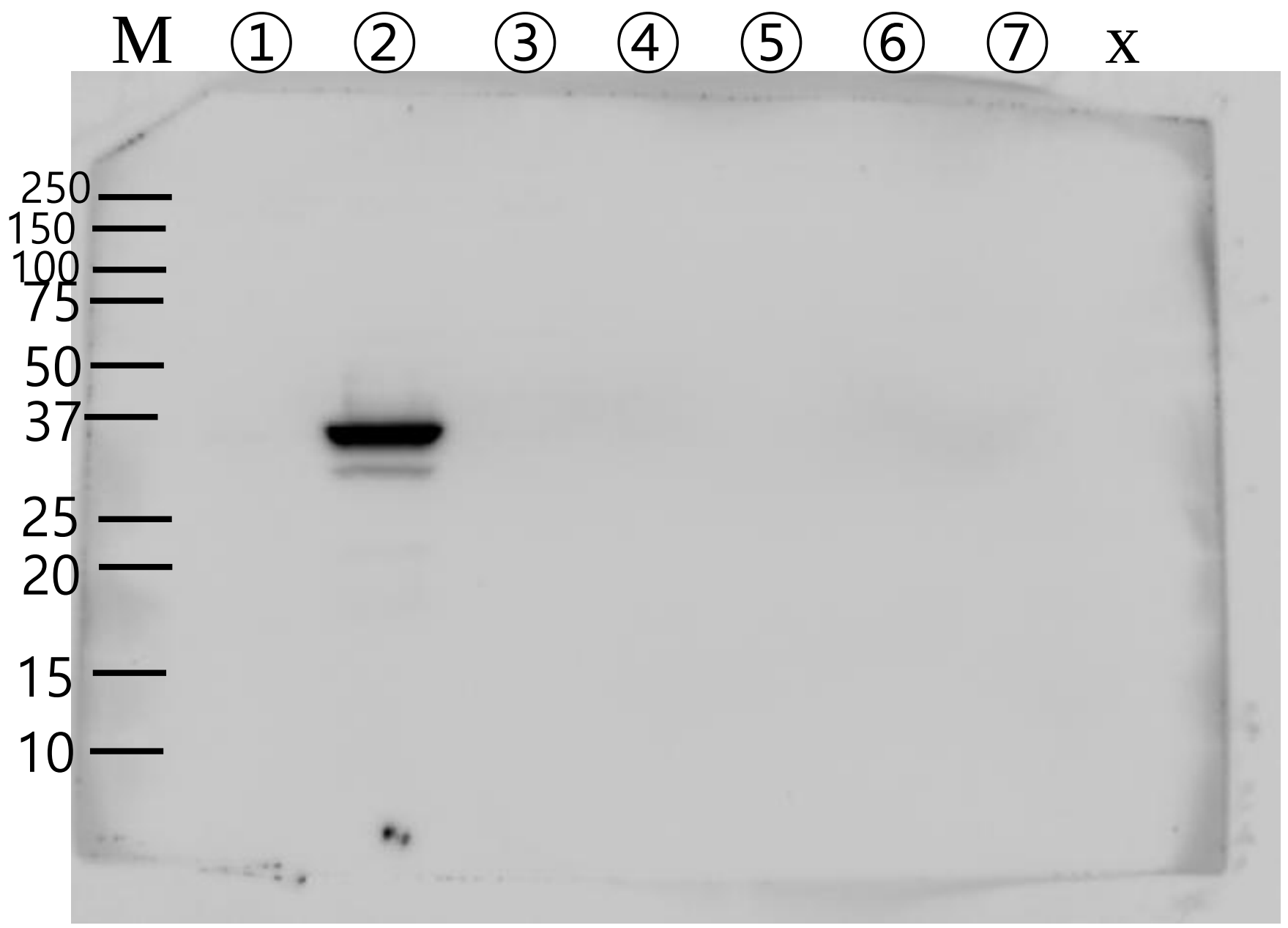

Technical replicate #2 (long exposure)

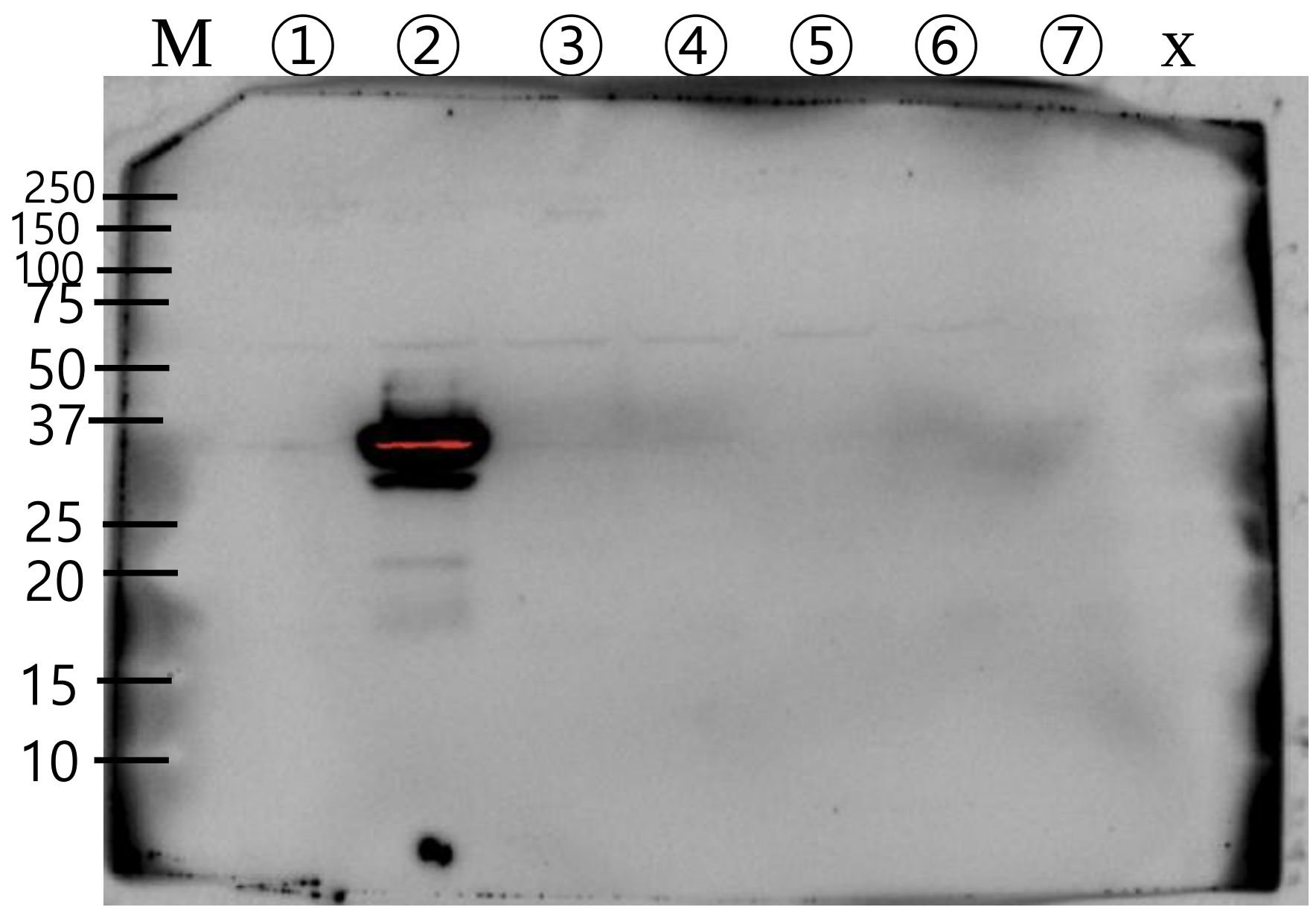

iNOS

Technical replicate #1 (used in Figure 6)

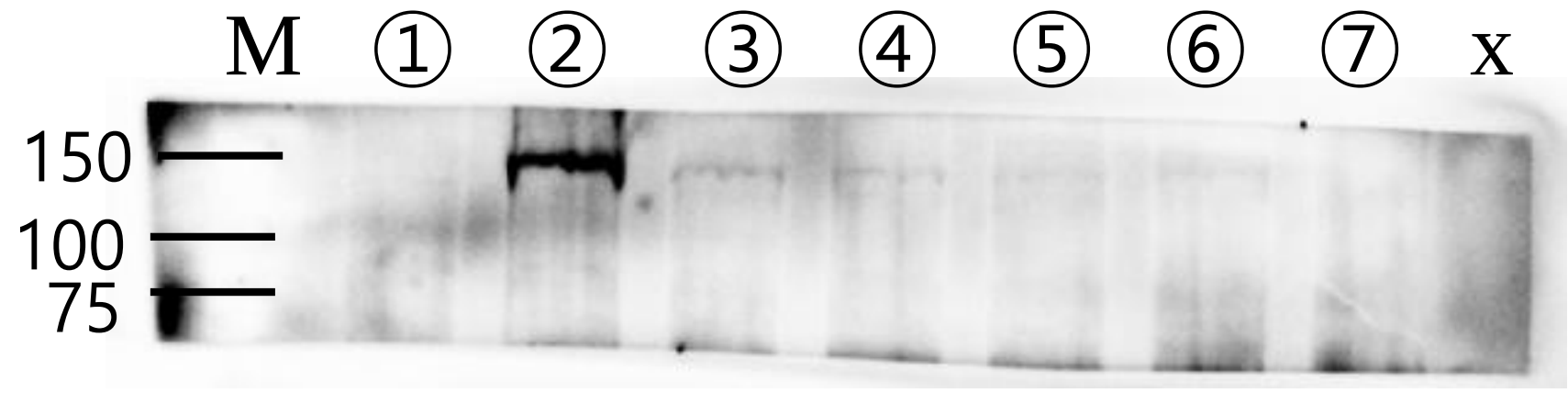

Technical replicate #2 (long exposure)

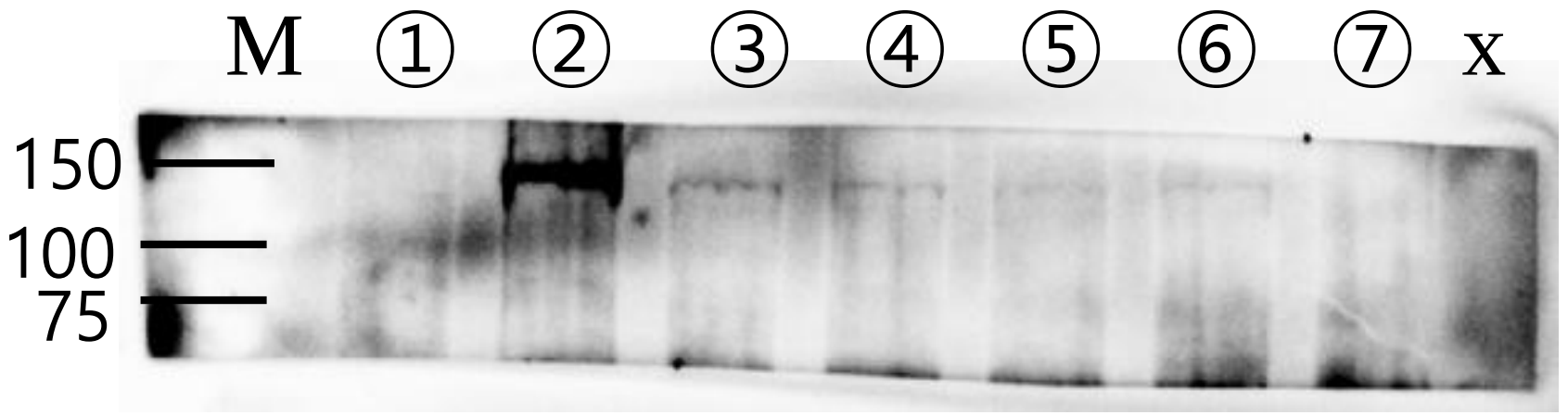

*** All blots are original, full-length, and unprocessed images acquired directly after detection.**

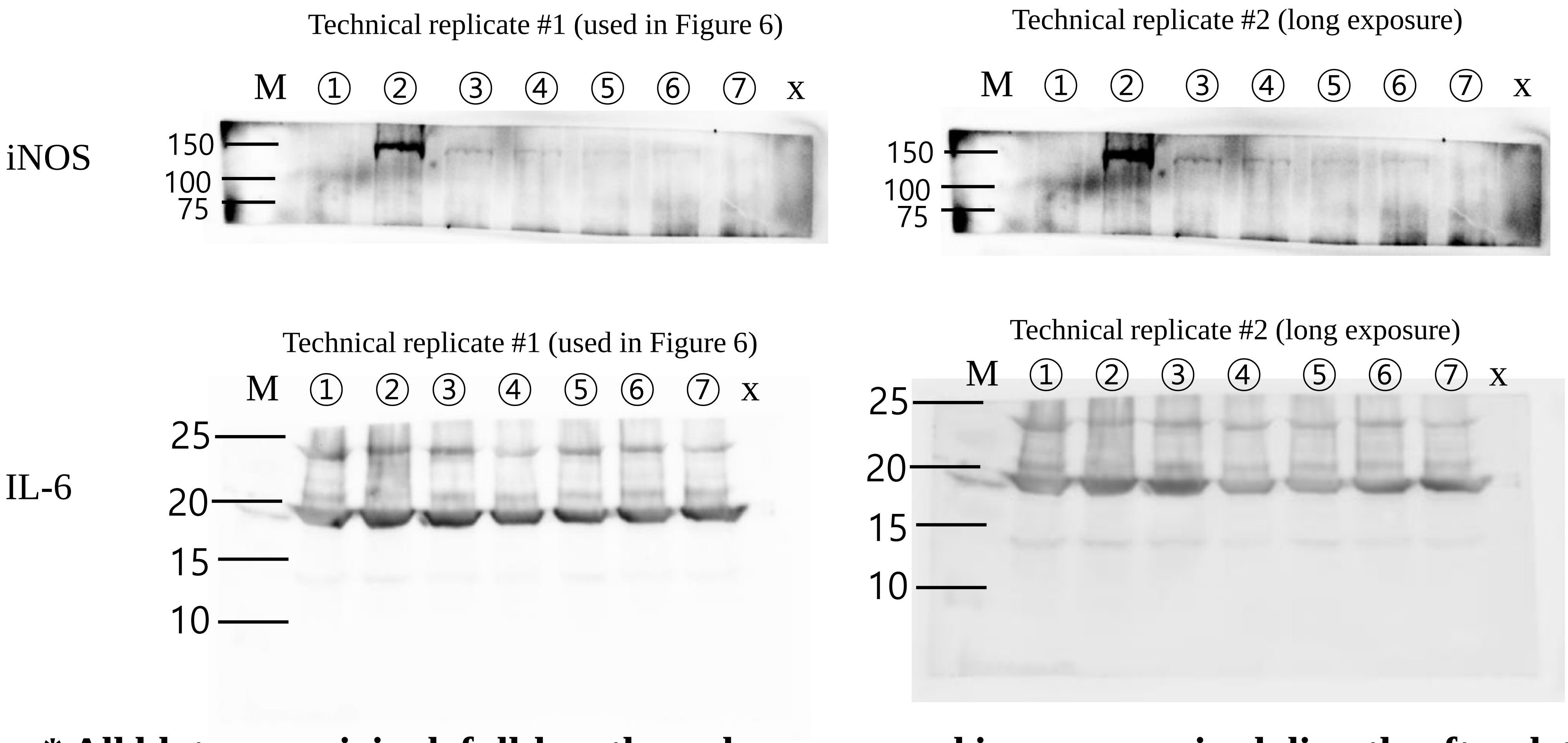

*** All blots are original, full-length, and unprocessed images acquired directly after detection.**

12hr

* Loading: ①M0 ②M1 ③ IS125 ④IS250 ⑤ IS125+vitD 30nM ⑥ IS250+vitD 30nM ⑦vitD 30nM

TGF-β1

Technical replicate #1 (used in Figure 6)

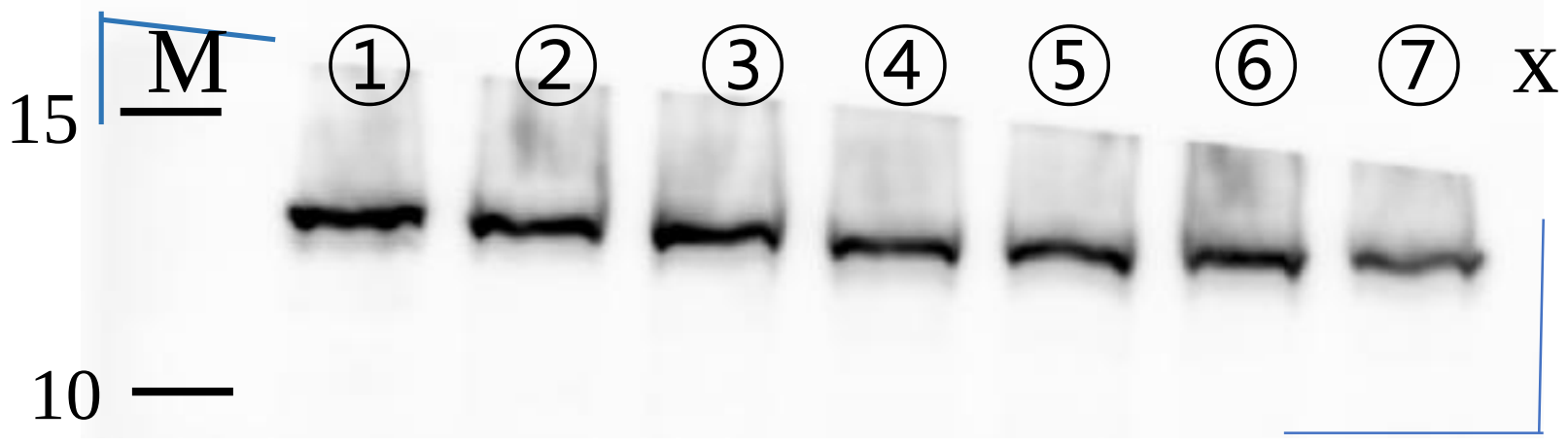

Technical replicate #2 (short exposure)

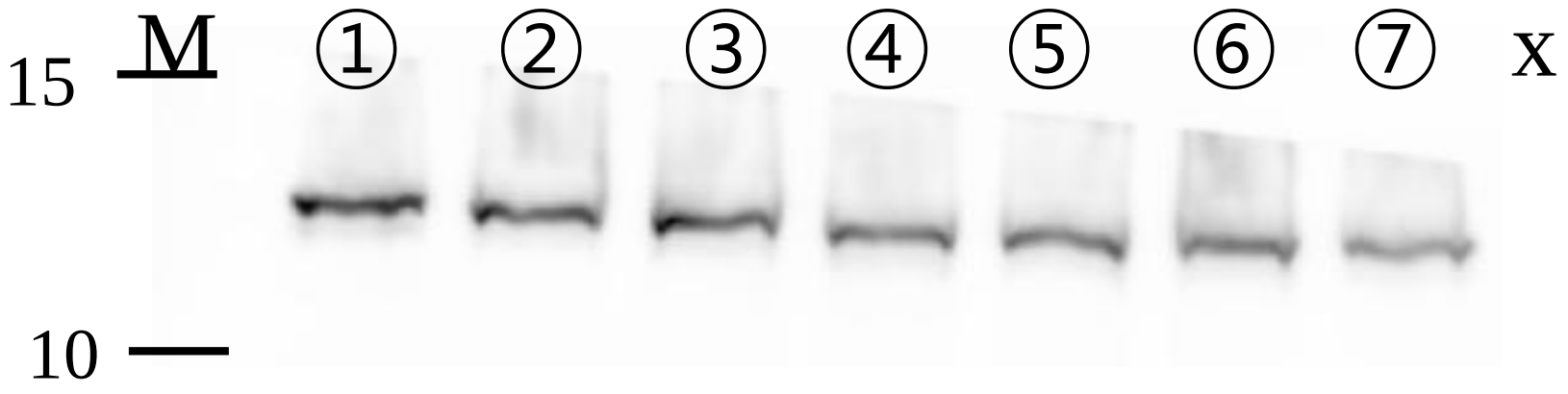

ABCA1

Technical replicate #1 (used in Figure 6)

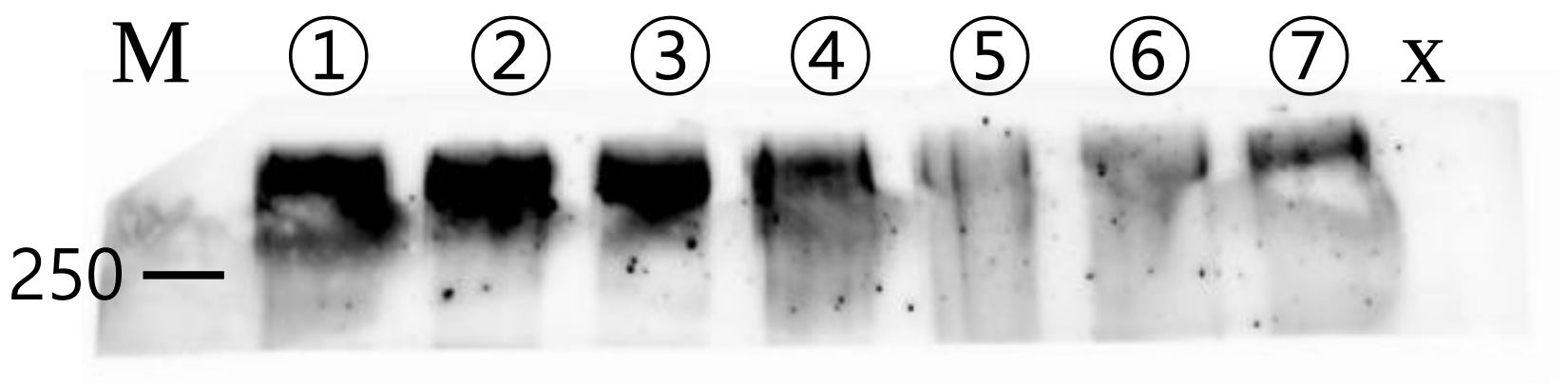

Technical replicate #2 (short exposure)

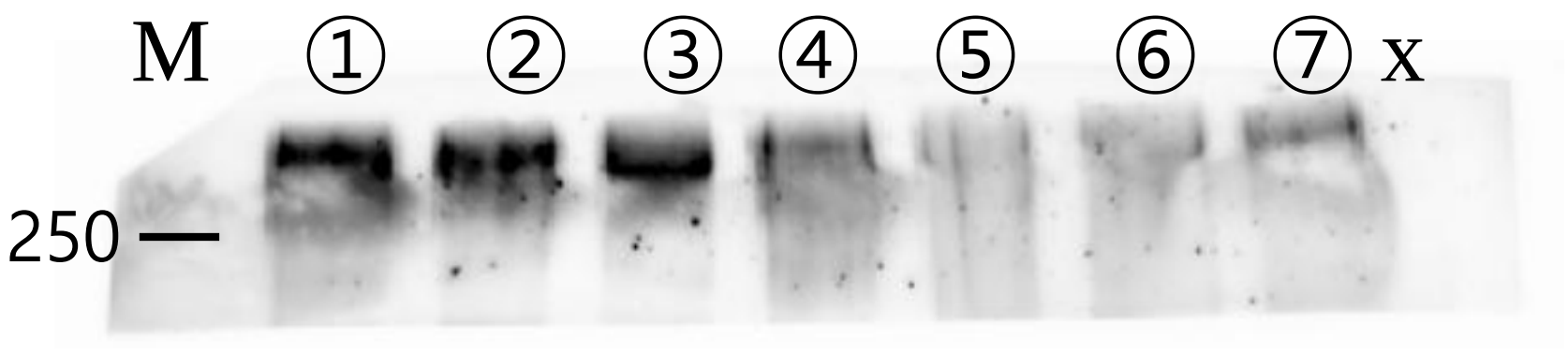

12hr

* Loading: ①M0 ②M1 ③ IS125 ④IS250 ⑤ IS125+vitD 30nM ⑥ IS250+vitD 30nM ⑦vitD 30nM

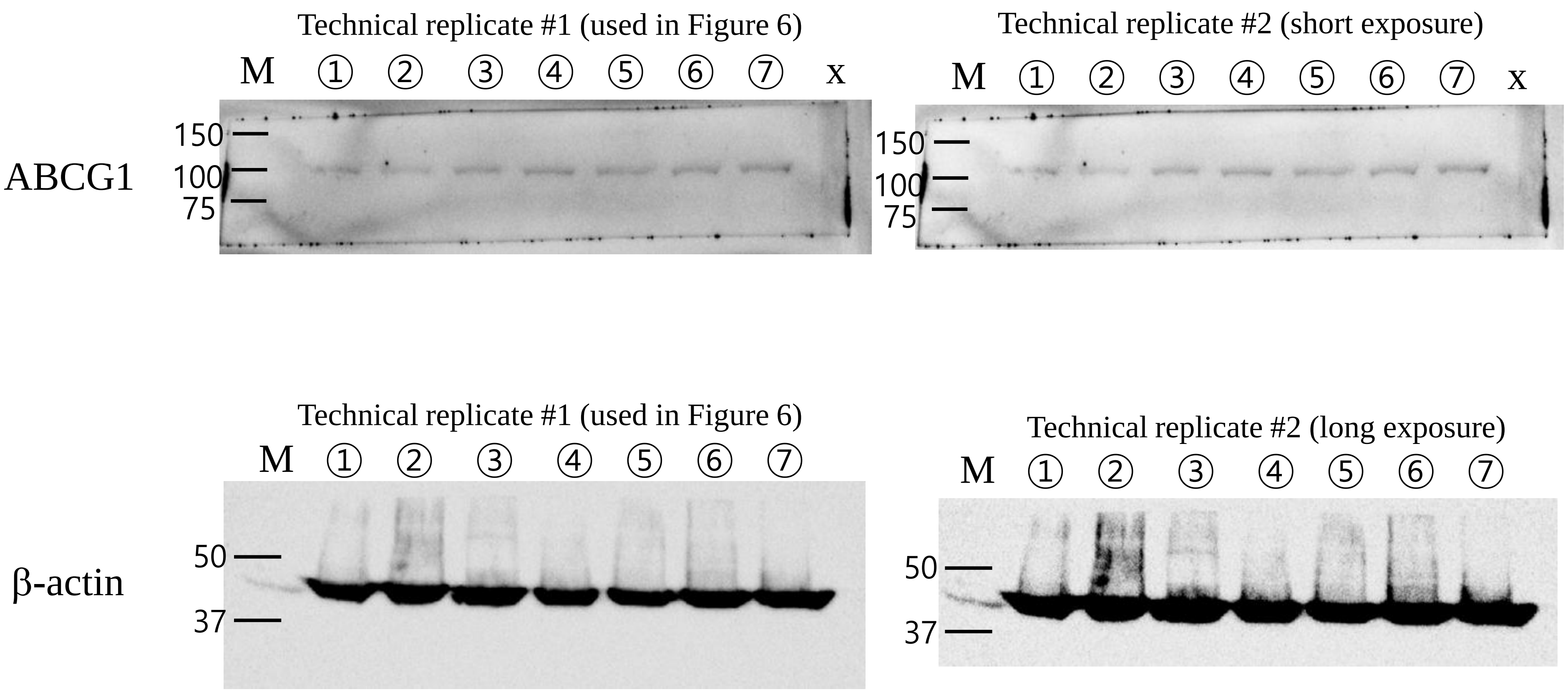

For β-actin, membrane edges are less discernible due to minimal background signal achieved under optimized exposure conditions, while band integrity and lane continuity are clearly preserved.

24hr

* Loading: ①M0 ②M1 ③ IS125 ④IS250 ⑤ IS125+vitD 30nM ⑥ IS250+vitD 30nM ⑦vitD 30nM

Technical replicate #1 (used in Figure 6)

IL-1β

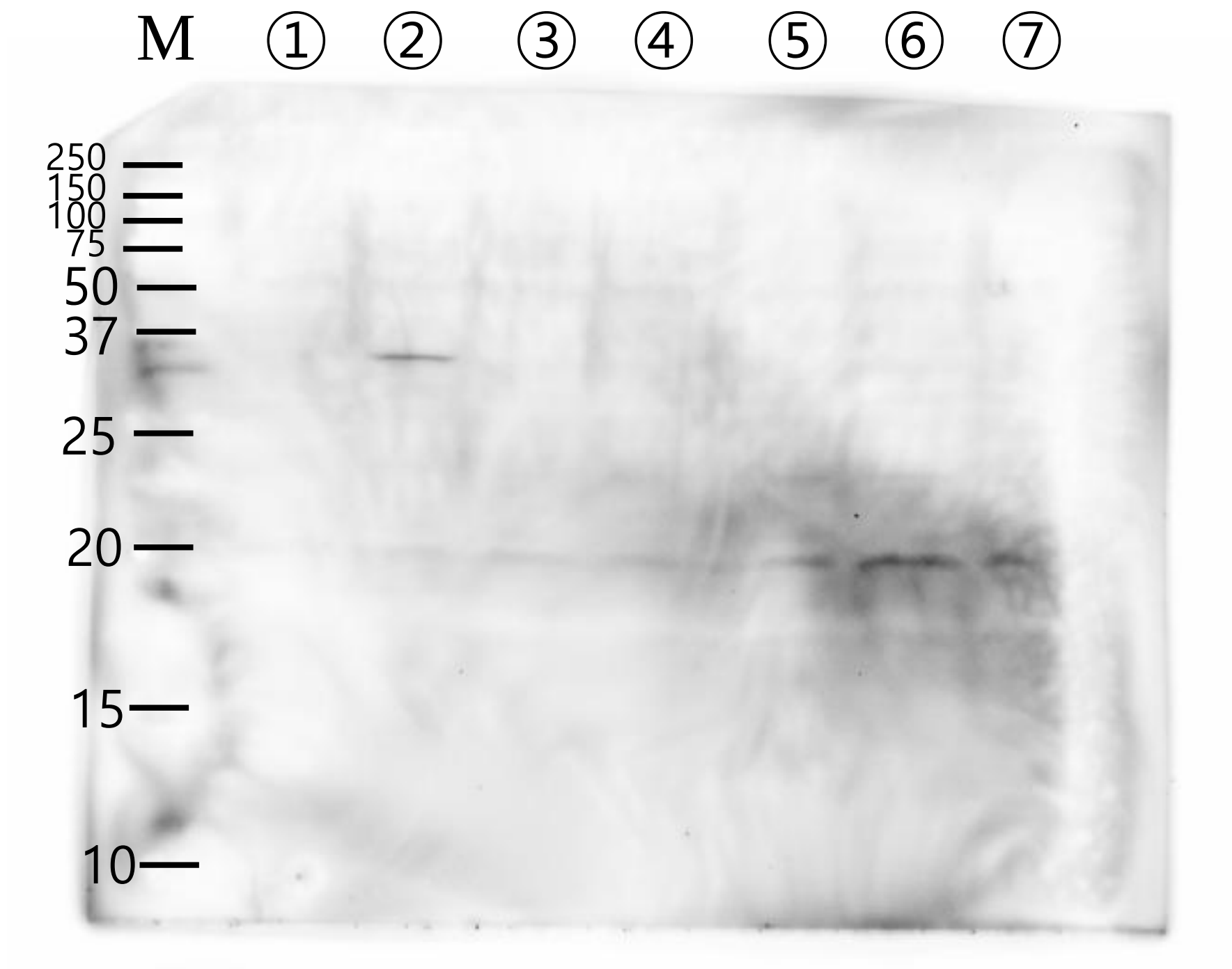

Technical replicate #2 (short exposure)

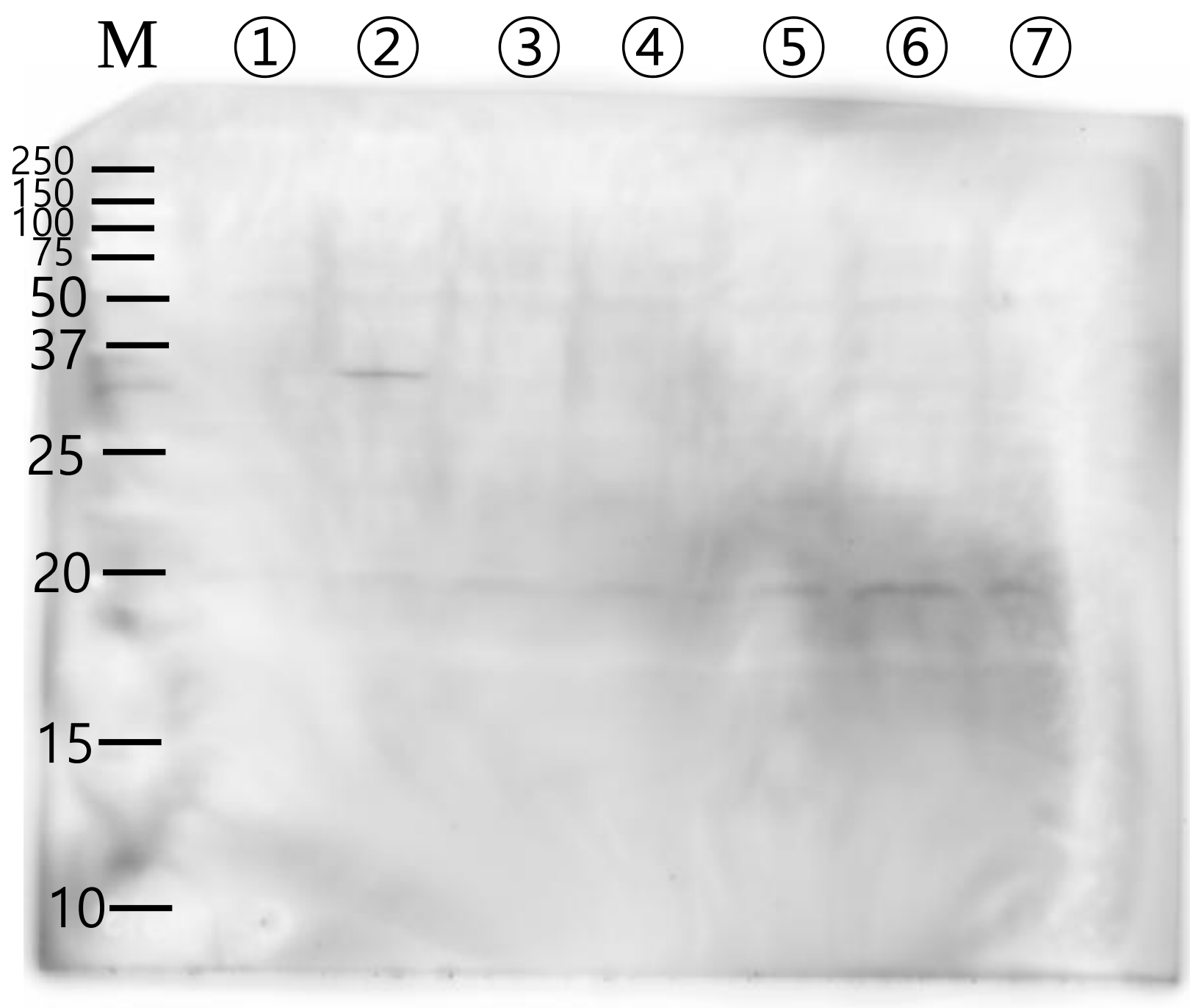

24hr

* Loading: ①M0 ②M1 ③ IS125 ④IS250 ⑤ IS125+vitD 30nM ⑥ IS250+vitD 30nM ⑦vitD 30nM

iNOS

Technical replicate #1 (used in Figure 6)

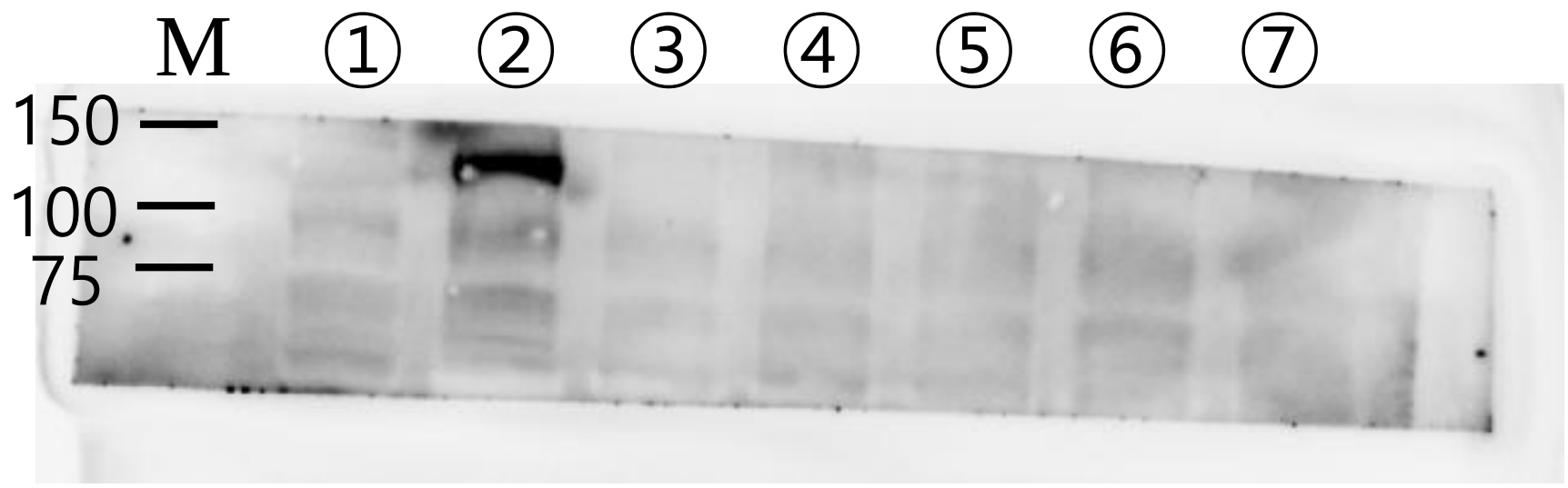

Technical replicate #2 (long exposure)

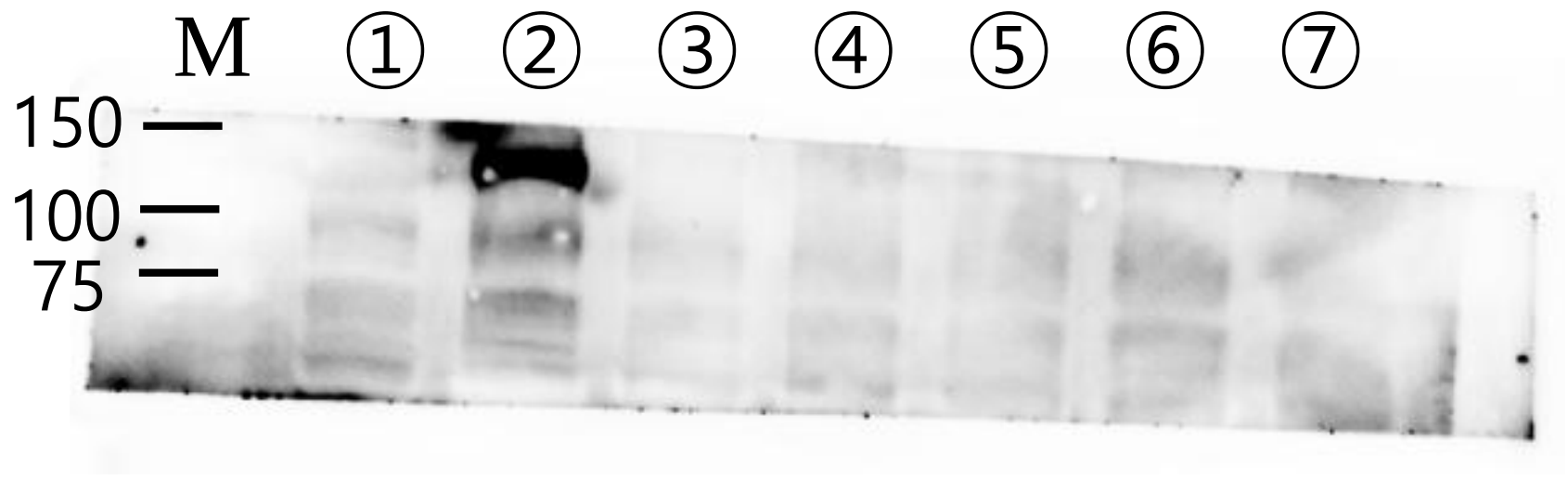

IL-6

Technical replicate #1 (used in Figure 6)

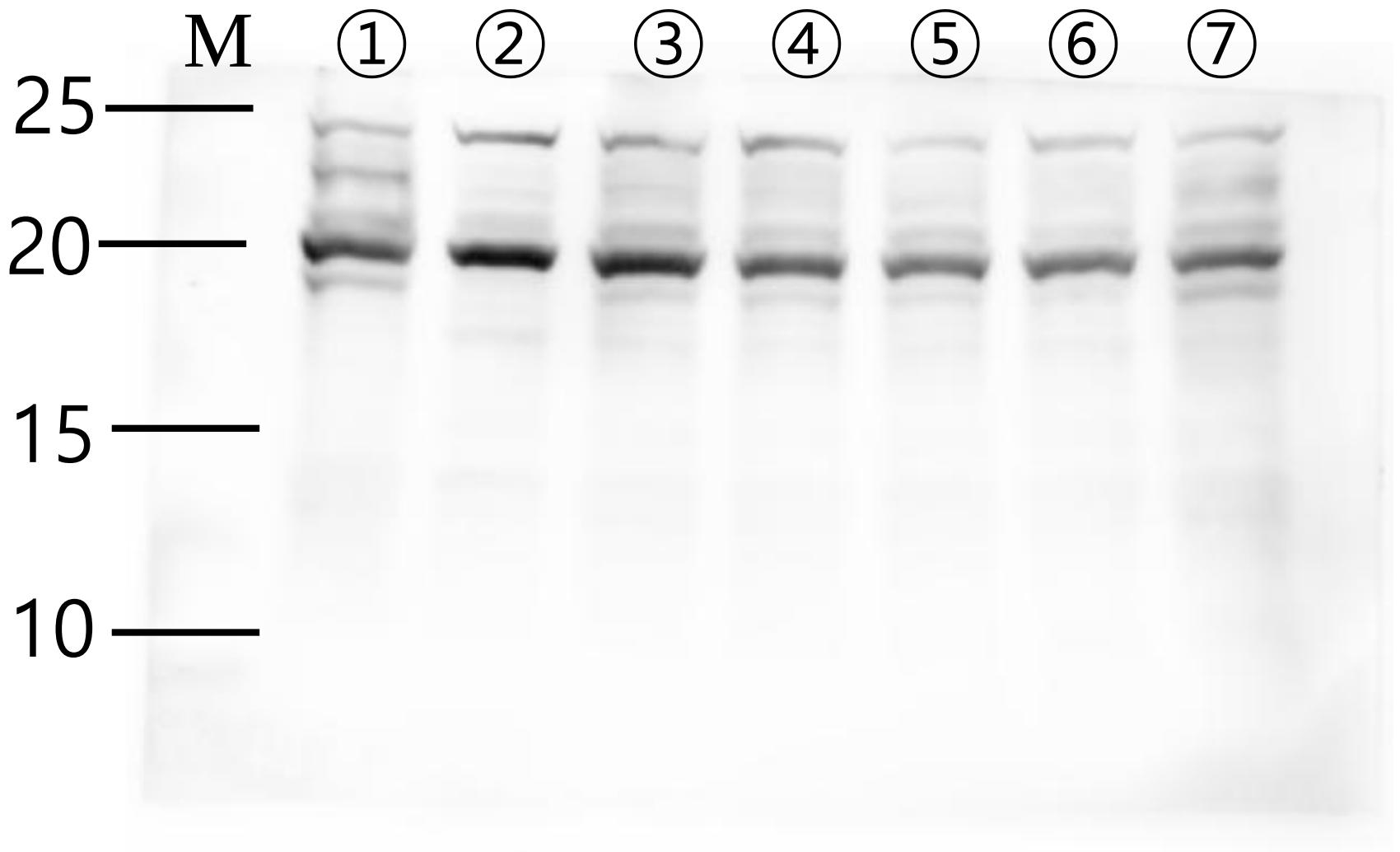

Technical replicate #2 (long exposure)

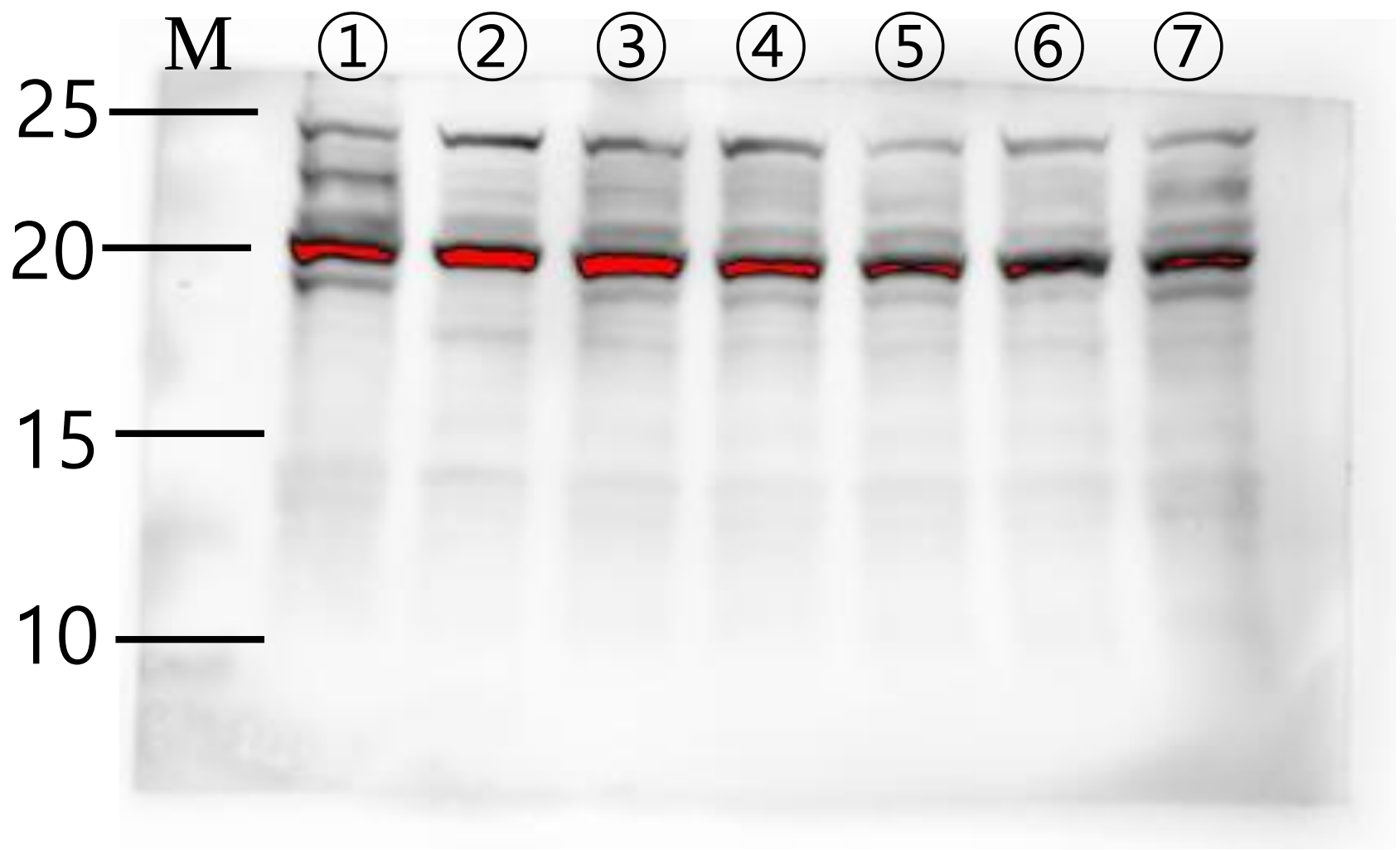

24hr

* Loading: ①M0 ②M1 ③ IS125 ④IS250 ⑤ IS125+vitD 30nM ⑥ IS250+vitD 30nM ⑦vitD 30nM

TGF-β1

Technical replicate #1 (used in Figure 6)

M ① ② ③ ④ ⑤ ⑥ ⑦ x

15

10

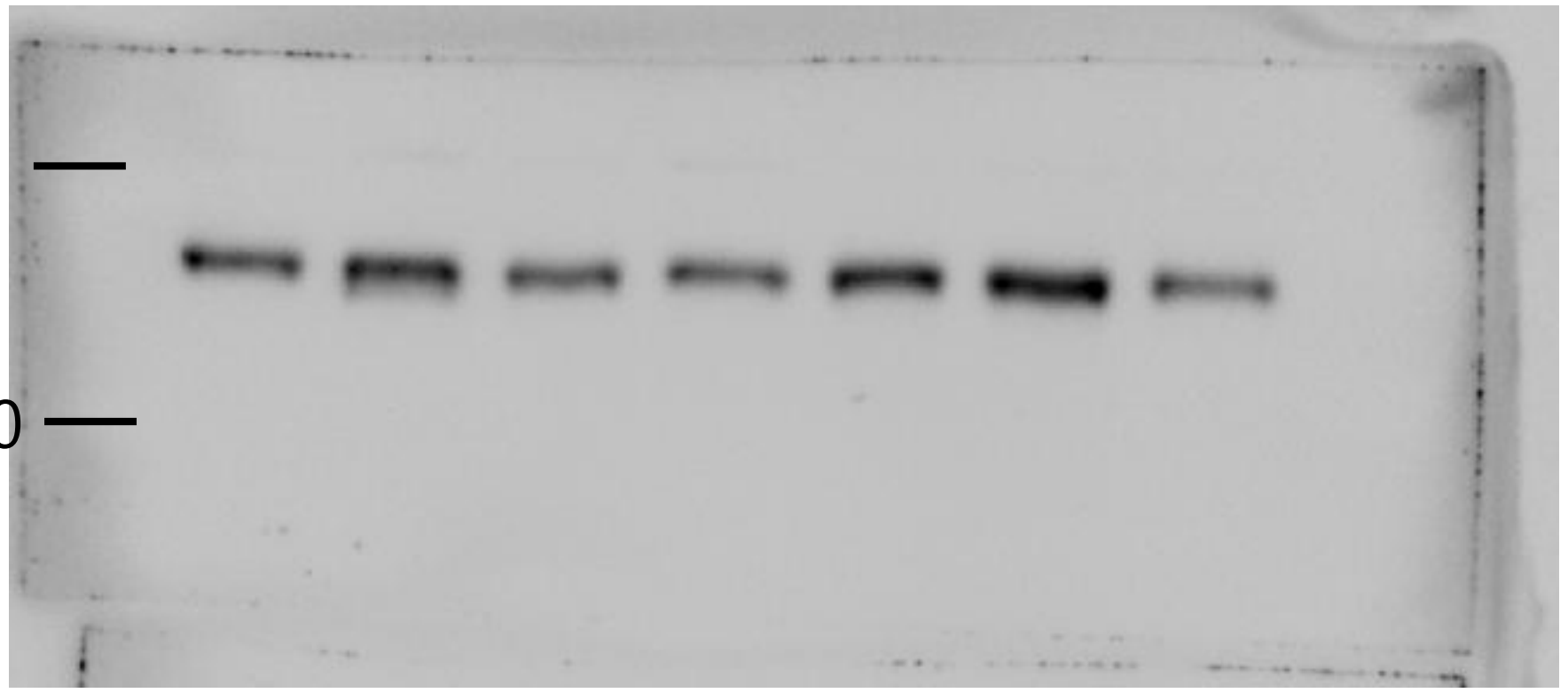

Technical replicate #2 (short exposure)

M ① ② ③ ④ ⑤ ⑥ ⑦ x

15

10

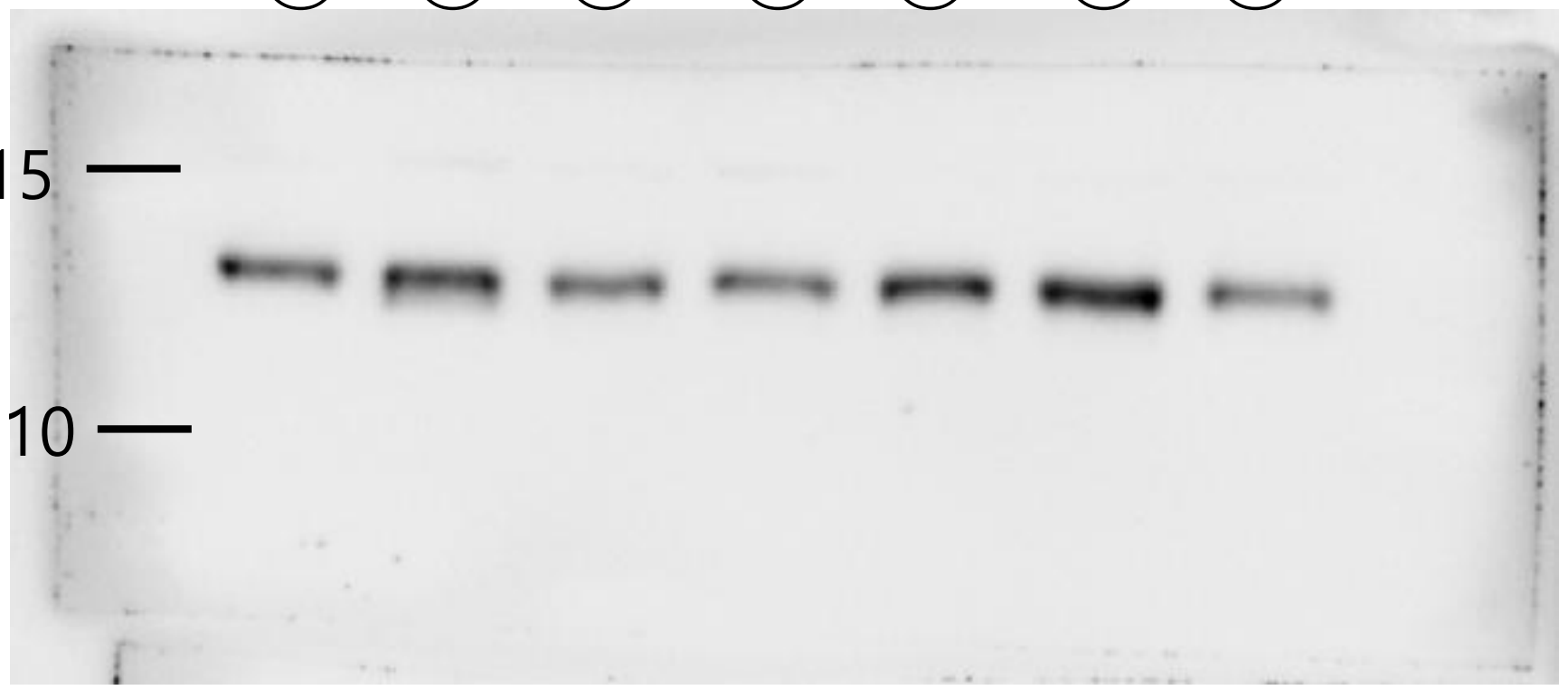

ABCA1

Technical replicate #1 (used in Figure 6)

M ① ② ③ ④ ⑤ ⑥ ⑦ x

250 kDa

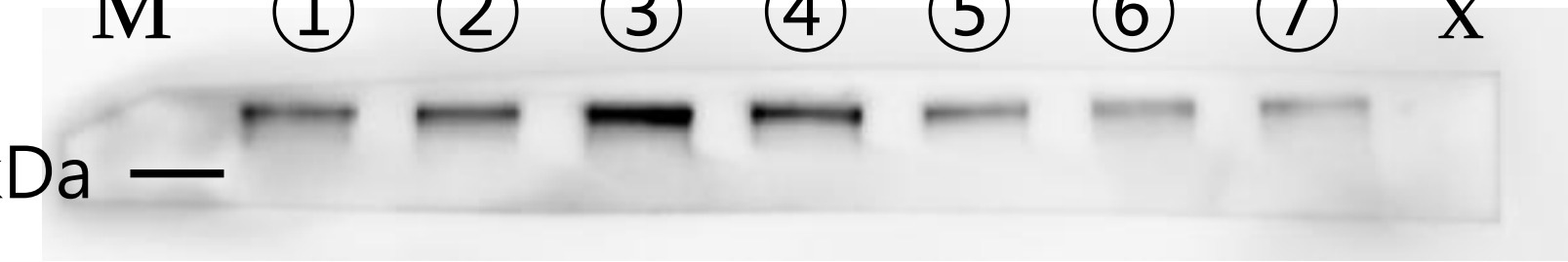

Technical replicate #2 (short exposure)

M ① ② ③ ④ ⑤ ⑥ ⑦ x

250 kDa

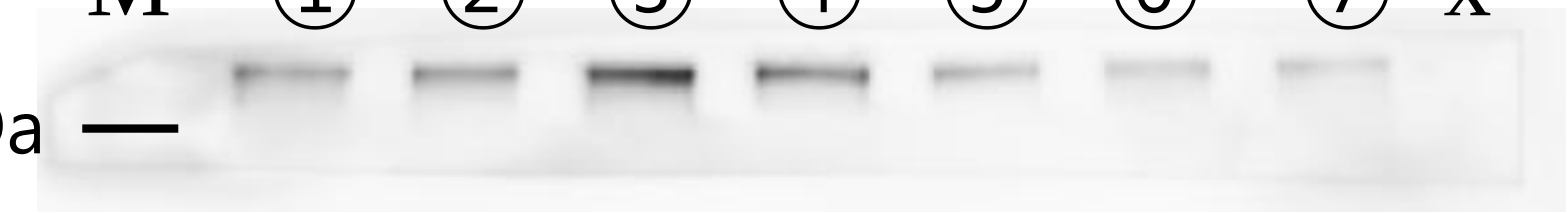

24hr

* Loading: ①M0 ②M1 ③ IS125 ④IS250 ⑤ IS125+vitD 30nM ⑥ IS250+vitD 30nM ⑦vitD 30nM

ABCG1

Technical replicate #1 (used in Figure 6)

M ① ② ③ ④ ⑤ ⑥ ⑦ x

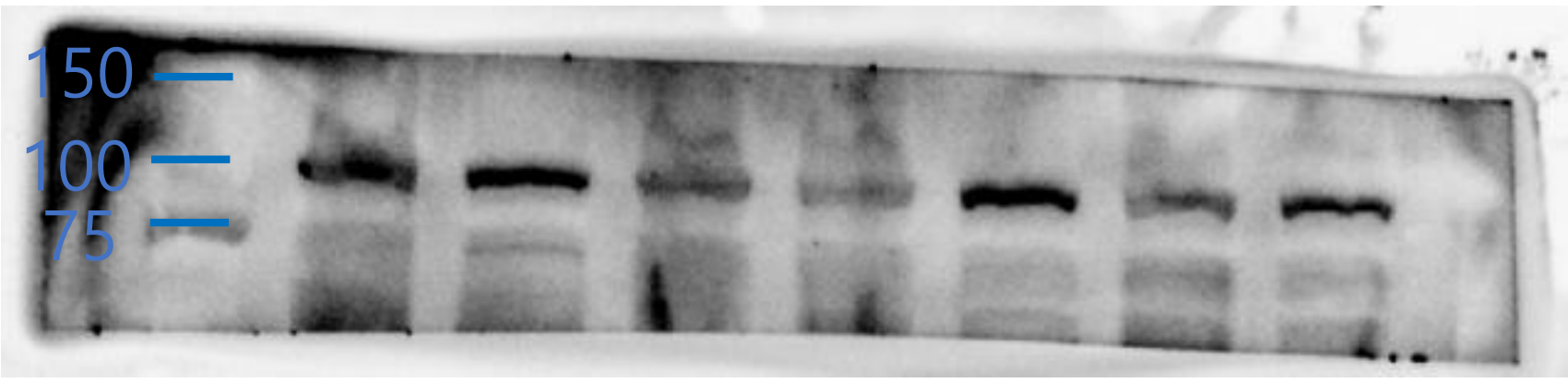

Technical replicate #2 (short exposure)

M ① ② ③ ④ ⑤ ⑥ ⑦ x

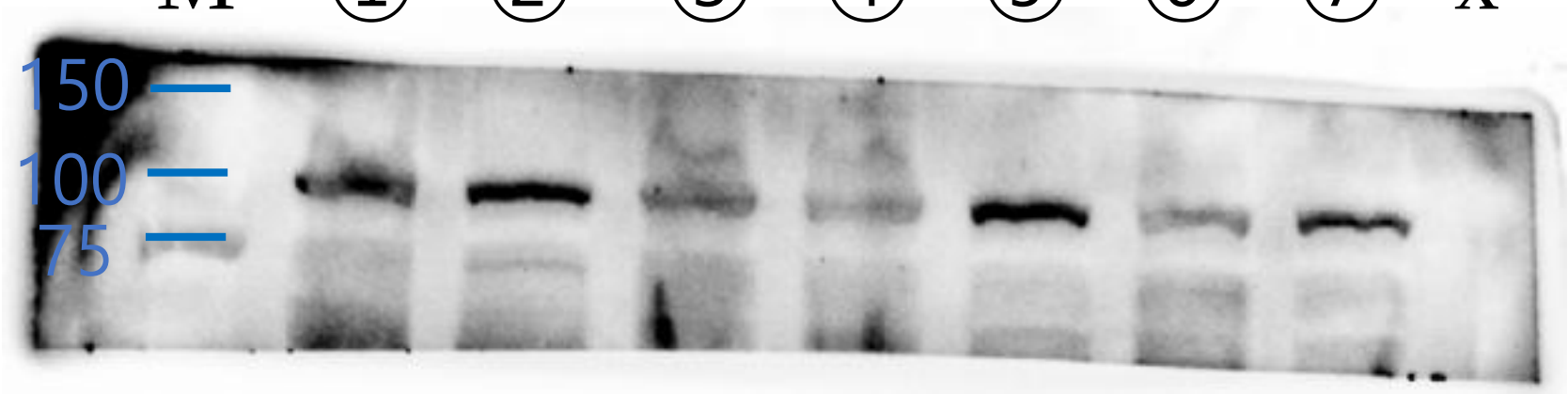

β-actin

Technical replicate #1 (used in Figure 6)

M ① ② ③ ④ ⑤ ⑥ ⑦ x

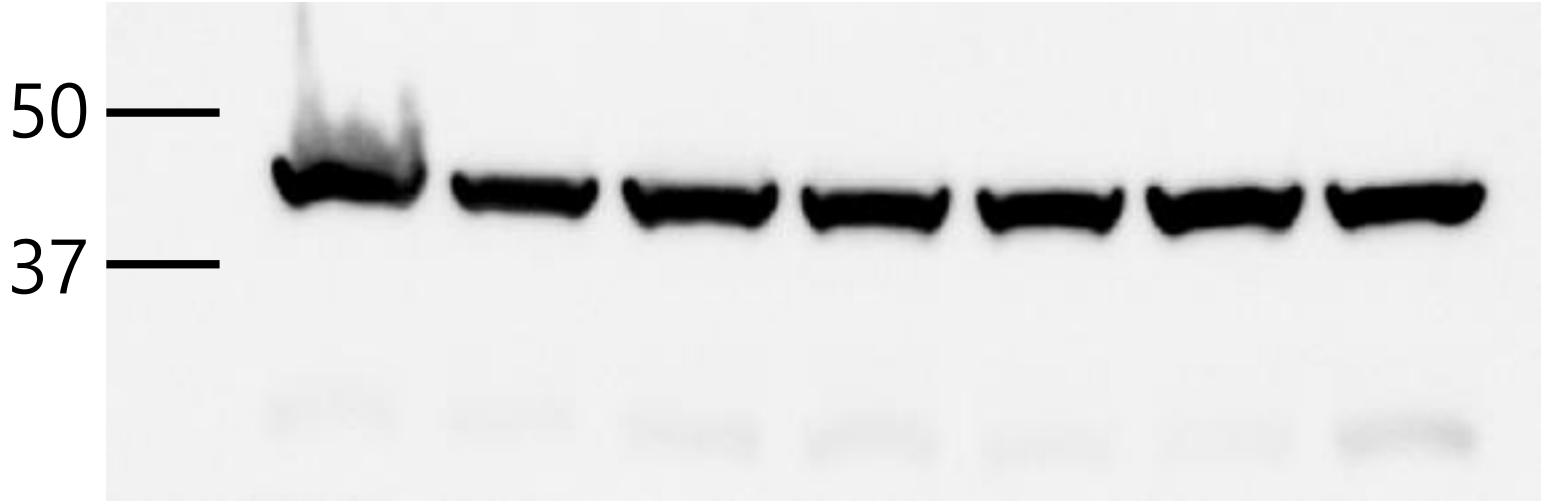

Technical replicate #2 (long exposure)

M ① ② ③ ④ ⑤ ⑥ ⑦ x

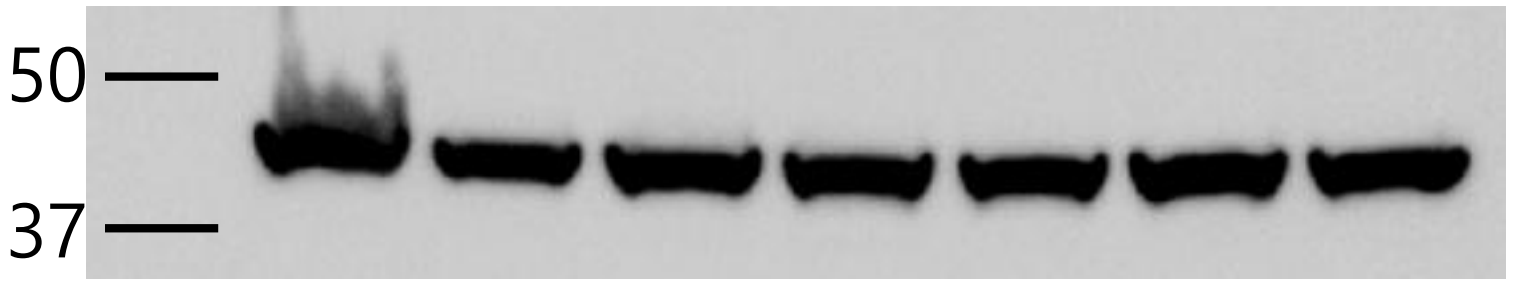

For β-actin, membrane edges are less discernible due to minimal background signal achieved under optimized exposure conditions, while band integrity and lane continuity are clearly preserved.
